# Mild‐Photothermal Effect Induced High Efficiency Ferroptosis‐Boosted‐Cuproptosis Based on Cu_2_O@Mn_3_Cu_3_O_8_ Nanozyme

**DOI:** 10.1002/advs.202303694

**Published:** 2023-10-11

**Authors:** Wei Chen, Wenyu Xie, Zhimin Gao, Chen Lin, Meiling Tan, Yaru Zhang, Zhiyao Hou

**Affiliations:** ^1^ Guangzhou Municipal and Guangdong Provincial Key Laboratory of Protein Modification and Degradation School of Basic Medical Sciences Guangzhou Medical University Guangzhou 511436 P. R. China

**Keywords:** cuproptosis, ferroptosis, mild‐photothermal effect, nanozyme, tumor microenvironment

## Abstract

A core‐shell‐structured Cu_2_O@Mn_3_Cu_3_O_8_ (CMCO) nanozyme is constructed to serve as a tumor microenvironment (TME)‐activated copper ionophore to achieve safe and efficient cuproptosis. The Mn_3_Cu_3_O_8_ shell not only prevents exposure of normal tissues to the Cu_2_O core to reduce systemic toxicity but also exhibits enhanced enzyme‐mimicking activity owing to the better band continuity near the Fermi surface. The glutathione oxidase (GSHOx)‐like activity of CMCO depletes glutathione (GSH), which diminishes the ability to chelate Cu ions, thereby exerting Cu toxicity and inducing cuproptosis in cancer cells. The catalase (CAT)‐like activity catalyzes the overexpressed H_2_O_2_ in the TME, thereby generating O_2_ in the tricarboxylic acid (TCA) cycle to enhance cuproptosis. More importantly, the Fenton‐like reaction based on the release of Mn ions and the inactivation of glutathione peroxidase 4 induced by the elimination of GSH results in ferroptosis, accompanied by the accumulation of lipid peroxidation and reactive oxygen species that can cleave stress‐induced heat shock proteins to compromise their protective capacity of cancer cells and further sensitize cuproptosis. CMCO nanozymes are partially sulfurized by hydrogen sulfide in the colorectal TME, exhibiting excellent photothermal properties and enzyme‐mimicking activity. The mild photothermal effect enhances the enzyme‐mimicking activity of the CMCO nanozymes, thus inducing high‐efficiency ferroptosis‐boosted‐cuproptosis.

## Graphical abstract

The Cu_2_O@Mn_3_Cu_3_O_8_ nanozyme exhibits excellent mimic enzyme activities due to better band continuity near the Fermi surface, which can be served as tumor microenvironment activated copper ionophore for achieving safe and efficient cuproptosis. More interestingly, cuproptosis can be sensitized with ferroptosis through cleaving protective heat shock proteins to enhance proteotoxicity, especially in inhibiting colorectal tumors with mild photothermal effect irradiation.

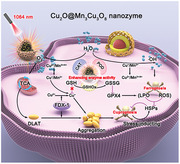

## 1 Introduction

Metal ions are essential for the normal activities of an organism and play important roles in various physiological processes.^[^ ^1^ ^]^ Due to their potency in trace amounts and high efficiency, the exploitation of metal ions has emerged as a breakthrough for affecting physiological functions. Metal ions can efficiently suppress the growth of cancer cells without susceptibility to drug resistance by triggering biocatalysis, disrupting the osmotic balance, affecting metabolism, interfering with signal transduction, and damaging DNA.^[^ ^2^ ^]^ Among the numerous metal ions, cell death induced by copper ions is unique and distinct from other known cell death pathways (including apoptosis, necrosis, pyroptosis, autophagy, and ferroptosis). According to the inceptive research by Tsvetkov et al., the binding of copper to lipoylated components in the tricarboxylic acid (TCA) cycle can induce the aggregation of lipoylated proteins and subsequent loss of iron–sulfur (Fe–S) cluster proteins, which causes cell death via cuproptosis due to proteotoxic stress.^[^ ^3^ ^]^ The two key regulators of cuproptosis are lipoylation and the upstream regulator, FDX1. Specifically, FDX1 reduces Cu^2+^ to the more toxic Cu^+^ and promotes the clustering of lipoylated proteins and enzymes (especially dihydrolipoamide *S*‐acetyltransferase, DLAT) involved in regulating the TCA cycle, whilst FDX1 results in the loss of Fe–S cluster proteins.^[^ ^4^ ^]^

Considering the potential mechanism of cuproptosis and the characteristics of the tumor microenvironment (TME), there are still four questions regarding the construction of copper ionophores that need to be addressed for the application of cuproptosis in tumor treatment. First, chelation between copper and reduced glutathione (GSH, highly expressed in the TME),^[^ ^5^ ^]^ which limits the binding of copper to the lipoylated components in the TCA, can inhibit the efficacy of cuproptosis. Tsvetkov et al. found that cuproptosis can upregulate stress‐induced heat shock proteins (HSPs), such as HSP70,^[^ ^3^ ^]^ thereby enhancing the stress capacity of cells, which in turn protects cellular proteins from stress‐induced denaturation, misfolding, and aggregation.^[^ ^6^ ^]^ Therefore, inhibiting cellular self‐protection by HSPs is the second issue to be addressed for applying cuproptosis in tumor treatment. Third, mitochondrial respiration involving cuproptosis requires the participation of oxygen (O_2_), whereas the TME is characterized by hypoxia,^[^ ^7^ ^]^ which can also limit the effect of cuproptosis. Finally, copper‐induced cell death is not specific to cancer cells as copper is equally lethal to normal cells. Thus, the rational design of TME‐activated nanoagents that serve as copper ionophores is significant for developing a responsive strategy for solving the above problems and achieving efficient and safe antitumor effects via cuproptosis.

In recent years, nanozymes that mimic natural enzymatic activity have become a frontier in nanomedicine research.^[^ ^8^ ^]^ Owing to the catalytic response of multivalent metal ions (e.g., Fe^2+/3+^, Cu^1+/2+^, and Mn^2+/4+^), inorganic nanozymes harboring metal ion redox couples that exhibit glutathione oxidase (GSHOx)‐like, peroxidase (POD)‐like, and catalase (CAT)‐like activities have shown remarkable efficacy in modulating the TME, thereby enhancing the therapeutic antitumor effects.^[^ ^9^ ^]^ GSHOx‐like activity decomposes overexpressed intercellular GSH in tumor cells,^[^ ^10^ ^]^ thus enhancing copper toxicity by effectively inhibiting the chelation of copper ions by GSH.^[^ ^11^ ^]^ Moreover, GSH depletion inactivates glutathione peroxidase 4 (GPX4), thereby upregulating lethal lipid peroxidation (LPO) and reactive oxygen species (ROS),^[^ ^12^ ^]^ which eventually induces ferroptosis. The generation of hydroxyl radicals (·OH) is augmented by catalyzing the decomposition of highly expressed hydrogen peroxide (H_2_O_2_) in the TME via POD‐like activity,^[^ ^13^ ^]^ which further consumes GSH, thereby enhancing copper toxicity and ferroptosis.^[^ ^14^ ^]^ A large amount of LPO and ROS can crosslink the primary amines of proteins, which destroys their structure and function, thus cleaving HSP70 and reducing the protective stress capacity of cancer cells.^[^ ^15^ ^]^ The CAT‐like activity of the nanozyme allows it to react with endogenous H_2_O_2_ to generate oxygen (O_2_),^[^ ^16^ ^]^ reversing the hypoxic tumor microenvironment, which enhances mitochondrial respiration and promotes cuproptosis. Some inorganic nanozymes with strong absorption in the near‐infrared (NIR) region exhibit NIR‐triggered photothermal effects for sensitizing enzyme‐mimicking activity, which further enhances the therapeutic efficacy as a catalytic antitumor agent.^[^ ^17^ ^]^ More importantly, artificial nanozymes with various structures (including porous, hollow, and core‐shell) can undergo TME‐activated reactions and decomposition to achieve TME‐responsive anti‐tumor cuproptosis with low cytotoxicity to normal tissues.^[^ ^18^ ^]^

**Scheme 1:**
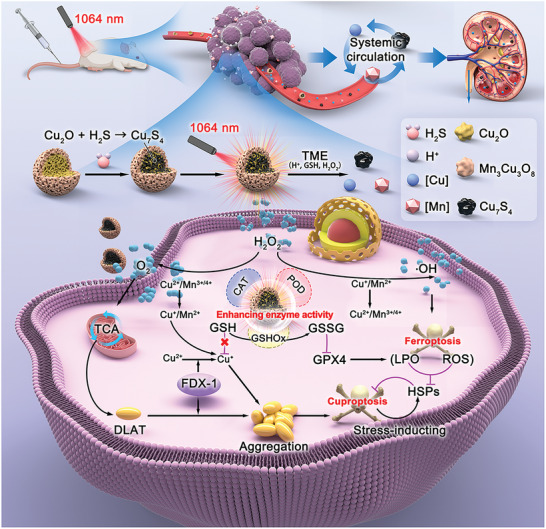
The mechanism of high efficiency ferroptosis‐boosted‐cuproptosis induced by mild‐photothermal effect based on Cu_2_O@Mn_3_Cu_3_O_8_ nanozymes for colorectal cancer therapy.

Cu_2_O, a semiconductor oxide containing monovalent copper ions (Cu^+^), can generate divalent copper ions (Cu^2+^) and elemental copper (Cu) by disproportionation reactions in an acidic environment,^[^ ^19^ ^]^ and thus can serve as a potential copper ionophore for TME‐activated cuproptosis. More importantly, Cu_2_O nanospheres can be used as matrix materials for constructing uniquely structured TME‐responsive nanozymes.^[^ ^20^ ^]^ However, the strong cytotoxicity of Cu_2_O in normal cells limits its biomedical applications. MnO_x_ nanomaterials exhibit GSHOx‐like,^[^ ^21^ ^]^ POD‐like,^[^ ^22^ ^]^ and CAT‐like^[^ ^23^ ^]^ enzyme‐mimicking activity in the TME, while avoiding potential long‐term toxicity in vivo by TME‐responsive catabolism.^[^ ^24^ ^]^ Thus, based on the characteristics of these two semiconductor oxides, the construction and design of TME‐responsive manganese–copper oxide nanozymes, which possess multiple functions for Cu‐ion delivery and mimic enzyme activities, is an innovative strategy for achieving safe and efficient cuproptosis. Herein, we report multifunctional Cu_2_O@Mn_3_Cu_3_O_8_ core‐shell nanozymes for NIR‐triggered high‐efficiency ferroptosis‐boosted‐cuproptosis, for treating colorectal tumors (**Scheme** **1**). The Mn_3_Cu_3_O_8_ shell and Mn_5_O_8_ shell are generated on the surface of Cu_2_O by using KMnO_4_ as the Mn source. In this process, polyvinylpyrrolidone (PVP) and bovine serum albumin (BSA) are respectively used as protective agents to avoid complete decomposition of Cu_2_O by the strongly oxidizing KMnO_4_. The Mn_3_Cu_3_O_8_ shell and Mn_5_O_8_ shell prevent the exposure of normal tissues to Cu_2_O prior to reaching the tumor lesion, thus reducing systemic toxicity. Moreover, the Mn_3_Cu_3_O_8_ shell exhibits enhanced enzyme‐mimicking activity owing to the better band continuity near the Fermi surface compared to that of the Mn_5_O_8_ shell. More interestingly, the diffusion of endogenous hydrogen sulfide (H_2_S) from the colorectal environment into the porous structure of Cu_2_O@Mn_3_Cu_3_O_8_ induces partial transformation of the metal‐semiconductor oxides to metal‐semiconductor sulfides having excellent photothermal effects,^[^ ^25^ ^]^ which further enhances the enzyme‐mimicking activity of Cu_2_O@Mn_3_Cu_3_O_8_ under NIR irradiation.

After entering the colorectal TME, Cu^2+^ ions are released from the Cu_2_O@Mn_3_Cu_3_O_8_ nanozymes, achieving enhanced cuproptosis through a multipronged strategy in a single system. The Cu^+^/Cu^2+^ and Mn^2+^/Mn^3+^/Mn^4+^ redox couples in the Cu_2_O@Mn_3_Cu_3_O_8_ nanozyme react with the overexpressed GSH and H_2_O_2_ in the TME to generate GSSG and O_2_ via GSHOx‐like‐ and CAT‐like activities, respectively. Relying on the dual strategy of avoiding chelation with GSH and enhancing mitochondrial respiration, the Cu toxicity is enhanced, leading to proteotoxic stress‐induced cancer cell death. Proteotoxic stress can upregulate the expression of HSP70 in cancer cells to enhance their stress capacity, thereby initiating a cellular self‐protection pathway to mitigate the damage caused by copper ion toxicity. The POD‐like activity of the nanozymes and Fenton‐like reaction of the released Mn ions catalyze the decomposition of overexpressed H_2_O_2_ in the TME to produce ·OH, which further depletes GSH and inactivates GPX4, thereby enabling ferroptosis. The large amount of LPO and ROS produced by ferroptosis in cancer cells depletes the stress protein HSP70 to relieve the limitation of ferroptosis. Partially sulfurized Cu_2_O@Mn_3_Cu_3_O_8_ undergoes moderate photothermal heating (under 1064 nm laser irradiation, less than 43°C, without thermal injury to normal tissues) and can thus significantly enhance the enzyme‐mimicking activity, thus realizing ferroptosis‐boosted high‐efficiency cuproptosis induced by a mild‐photothermal effect. After therapy, the Cu_2_O@Mn_3_Cu_3_O_8_ nanozymes effectively react with H_2_S in an acidic environment and are decomposed into Cu ions, Mn ions, and ultrasmall Cu_7_S_4_ nanoparticles, which can be effectively eliminated from the body in the urine via the renal filtration system, preventing long‐term retention of inorganic nanoparticles in the body. Therefore, this multi‐pronged strategy for enhancing cuproptosis is a safe and feasible treatment that can be applied in cancer therapy.

## 2 Results and Discussion

**Figure 1:**
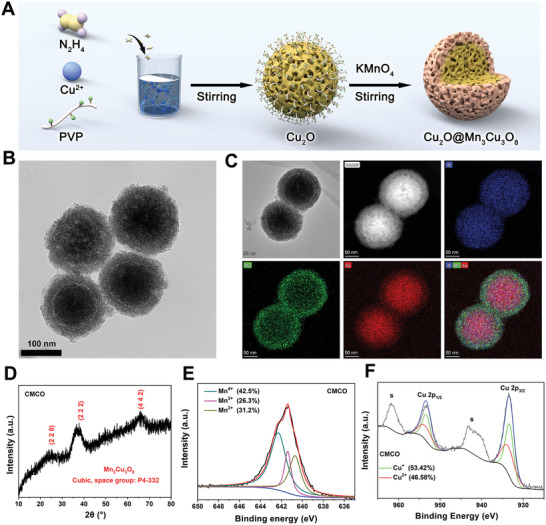
A) Schematic illustration of the synthesis process of CMCO. B) TEM image of CMCO. C) Elemental mapping of O, Mn and Cu of CMCO. D) XRD pattern of CMCO. The XPS high‐resolution scans of E) Mn 2p and F) Cu 2p in CMCO.

Cu_2_O@Mn_3_Cu_3_O_8_ (CMCO) core‐shell nanozymes were fabricated using cubic‐phase Cu_2_O nanospheres (Figures S1 and S2, Supporting Information) as templates in a KMnO_4_ hydrolysis solution. The Mn_3_Cu_3_O_8_ shell was generated in situ on the surface of Cu_2_O with the assistance of an appropriate PVP additive (**Figure** **1**A). In this synthesis strategy, PVP not only acts as a surfactant in the preparation of Cu_2_O, but also serves as a protective agent that prevents the complete decomposition of Cu_2_O by KMnO_4_ during the formation of the core‐shell structure. The reaction of Cu_2_O with KMnO_4_ for 2 h generated a core‐shell nanozyme with the largest shell thickness and best stability in the presence of NaHS (Figures S3 and S4, Supporting Information); this sample was selected for further evaluation. The transmission electron microscopy (TEM) images (Figure 1B) show that the surface of Cu_2_O was coated with a porous shell layer in situ after the reaction of Cu_2_O with KMnO_4_. The SEM images (Figure S5, Supporting Information) indicated that CMCO was structurally stable over time in different solutions and also demonstrated the stability of the Mn_3_Cu_3_O_8_ layer. The nitrogen adsorption isotherms of the CMCO nanozymes indicated the presence of micropores and mesopores, which increased the specific surface area of the nanoparticles (Figure S6, Supporting Information). The low‐magnification high‐angle annular dark‐field scanning TEM (HAADF–STEM) and elemental mapping images (Figure 1C; Figure S7, Supporting Information) show that Cu, Mn, and O were distributed in different parts of the core‐shell structure. Oxygen was homogeneously distributed throughout the core and shell, Mn was mainly distributed in the shell, and Cu was distributed in both the core and shell. The ratio of elements in the shell (Figure S8 and Table S1, Supporting Information) and the X‐ray diffraction (XRD) pattern (Figure 1D) indicate that the Mn_3_Cu_3_O_8_ shell, assigned to the cubic crystal system (space group: P4‐332), was formed outside the Cu_2_O core (Cuprite, PDF#78‐2076). Thus, the core‐shell structure is recognized as Cu_2_O@Mn_3_Cu_3_O_8_ (CMCO). The dynamic light scattering (DLS) data (Figure S9, Supporting Information) indicated that CMCO presented good dispersity, with an average hydrodynamic diameter of 176.8±10.8 nm, which is larger than that of Cu_2_O 126.5±13.4 nm. The Mn_3_Cu_3_O_8_ shell was 25.7±0.7 nm thick (Figure S10, Supporting Information).

**Figure 2:**
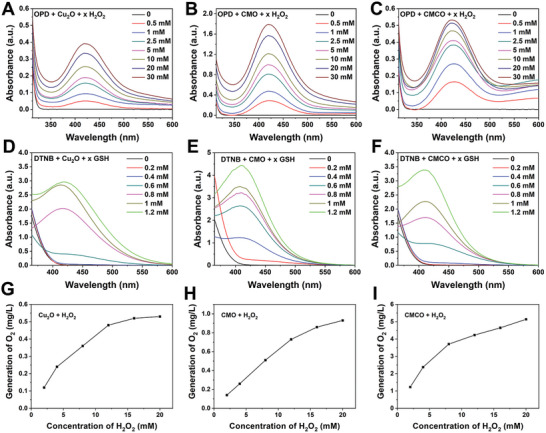
The OPD absorption curves due to ·OH generation by POD‐like activities of A) Cu_2_O, B) CMO, and C) CMCO (20 µg·mL^−1^) with H_2_O_2_ (from 0.5 to 30 mM) as a substrate and reaction for 300 s in PBS (pH 6.5). The DTNB absorption curves due to GSH consumption by GSHOx‐like activities of D) Cu_2_O, E) CMO, and F) CMCO (20 µg·mL^−1^) with GSH (from 0.2 to 1.2 mM) as a substrate and reaction for 300 s in PBS (pH 6.5). The O_2_ generation from H_2_O_2_ by CAT‐like activities of G) Cu_2_O, H) CMO, and I) CMCO (50 µg·mL^−1^) in PBS (pH 6.5) for 30 s.

The high‐resolution X‐ray photoelectron spectrum (XPS) of the Cu_2_O nanospheres in the Cu 2p region (Figure S11, Supporting Information) shows that Cu was present as Cu^+^ (86.74%) and Cu^2+^ (13.26%). The Cu^2+^ in Cu_2_O may be derived from the oxidation of Cu^+^ on the nanozyme surface during the preservation process or during detection. The Mn 2p and Cu 2p XPS profiles of CMCO (Figure S12, Supporting Information) demonstrate that Mn (Figure 1E) was present in the Mn^4+^ (42.5%)/Mn^3+^ (26%)/Mn^2+^ (31.2%) valence states and Cu (Figure 1F) was present as Cu^+^ (53.42%) and Cu^2+^ (46.58%). The higher ratio of Cu^2+^/Cu^+^ in CMCO confirmed that the outermost Cu in Cu_2_O was oxidized by KMnO_4_ in forming the Mn_3_Cu_3_O_8_ shell. Importantly, the Mn^2+^/Mn^3+^/Mn^4+^ and Cu^+^/Cu^2+^ redox couples provide immense potential for enzyme‐mimicking activity. Thus, we first studied the enzyme activity of the CMCO nanozymes in a mildly acidic TME (pH 6.5). Among the three nanozymes with core‐shell structures, CMCO obtained by the reaction of Cu_2_O with KMnO_4_ for 2 h showed the strongest POD‐like, GSHOx‐like, and CAT‐like activities (Figures S13 and S14, Supporting Information), which further confirmed the efficacy of the material. For comparison, another similar core‐shell structured Cu_2_O@Mn_5_O_8_ (CMO) nanocomposite was synthesized by the reaction of KMnO_4_ with BSA‐modified Cu_2_O (Figure S15, Supporting Information). First, *O*‐phenylenediamine (OPD) was used as a probe to determine the kinetic constants for the decomposition of H_2_O_2_ based on the POD‐like activities of Cu_2_O, CMO, and CMCO. As shown in **Figure** **2**A–C, the absorbance of oxOPD increased with the concentration of H_2_O_2_. The Michaelis–Menten constant (*K*_m_) and maximal reaction velocity (*V*_max_) of the POD‐like structures were calculated from the Lineweaver–Bruker plot. As shown in Figure S16 (Supporting Information), the *K*_m_ values for Cu_2_O, CMO, and CMCO were 0.301, 0.231, and 0.112 mM, and the *V*_max_ values were 6.86 × 10^−8^, 3.09 × 10^−7^, and 1.013× 10^−7^ M·s^−1^, respectively. These values indicate that CMO induces the fastest decomposition of H_2_O_2_ via POD‐like catalysis, whereas CMCO has the highest affinity for H_2_O_2_. Thus, 5,5′‐dithiobis‐(2‐nitrobenzoic acid) (DTNB) was used as a probe to determine the kinetic constants for GSH depletion (GSHOx‐like activity) by the nanozymes. The GSH that was not converted by the GSHOx‐like of nanozymes reacted with DTNB to generate TNB. Figure 2D–F shows the absorbance of TNB with different concentrations of GSH. Figure S17 (Supporting Information) presents the kinetic constants for GSH depletion (GSHOx‐like activity) by Cu_2_O, CMO, and CMCO. The respective *V*_max_ values were 16.16 × 10^−6^, 8.85 × 10^−6^, and 14.95 × 10^−6^ M·s^−1^, with corresponding *K*_m_ values of 0.47, 0.287, and 0.436 mM, respectively. Cu_2_O and CMCO exhibited similar GSHOx‐like reaction velocities, which were both higher than those of CMO. Finally, a dissolved oxygen analyzer was used to determine the kinetic constants for H_2_O_2_ decomposition (CAT‐like activity) by the nanozymes. As shown in Figure 2G–I, CMCO catalyzed the decomposition of H_2_O_2_ to generate significantly more O_2_ than that obtained with Cu_2_O and CMO. The values of *V*_max_ and *K*_m_ indicated that CMCO induced the fastest reaction and had the highest affinity (CAT‐like activity) for H_2_O_2_ (Figure S18, Supporting Information). In general, the catalytic activity of CMCO was higher than that of Cu_2_O after the formation of the core‐shell structure, and CMCO was superior to CMO in terms of the GSHOx‐like and CAT‐like activities.

**Figure 3:**
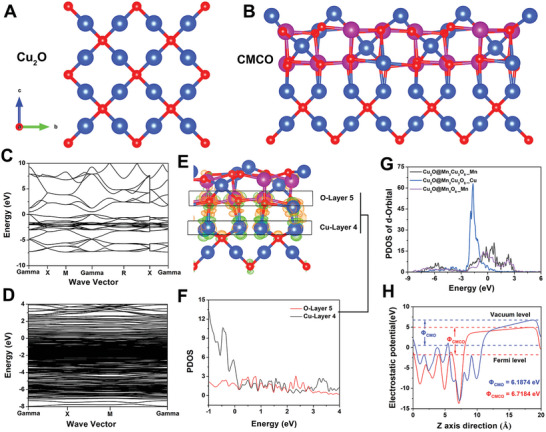
The structural models of A) Cu_2_O and B) CMCO nanozymes. The baseband calculation results of C) Cu_2_O and D) CMCO nanozymes. E) The differential charge density of CMCO nanozymes. F) The atomic densities of states of the O‐Layer 5 and Cu‐Layer 4 of CMCO nanozymes. G) The d‐orbital densities of states of Mn_5_O_8_ shell of CMO and Mn_3_Cu_3_O_8_ shell of CMCO. H) The electrostatic potentials of CMO and CMCO nanozymes.

To determine the effectiveness of the CMCO nanozymes as enzyme mimics, theoretical calculations were performed. The structural models of the Cu_2_O and CMCO nanozymes are shown in **Figure** **3**A,B, respectively. To understand the origin of the enhanced catalytic performance of the CMCO nanozymes after Cu_2_O was oxidized to generate the Mn_3_Cu_3_O_8_ shell on the surface, the Fermi levels of Cu_2_O, Mn_3_Cu_3_O_8_, and CMCO were calculated (Figure 3C,D; Figures S19 and S20, Supporting Information). The large bandgap of Cu_2_O renders it a semiconductor with poor electrical conductivity. The band continuity of Mn_3_Cu_3_O_8_ is significantly better near the Fermi surface than that of Cu_2_O, which classifies the former as a conductor with strong electrical conductivity. More importantly, over a wider energy range (*E*_f_ −8 eV to *E*_f_ +8 eV), the band continuity of CMCO was even stronger, which indicates that the catalytic activity of the core‐shell‐structured CMCO is stronger than that of Cu_2_O or Mn_3_Cu_3_O_8_ alone. Figure 3E shows the differential charge density for CMCO. Charge transfer occurs from Cu in Cu_2_O to O near Mn_3_Cu_3_O_8_, and an interaction (Cu─O bond) occurs between the heterojunctions. The atomic densities of the O‐Layer 5 and Cu‐Layer 4 states of the CMCO nanozymes were analyzed, as shown in Figure 3F. The PDOS values were clearly coupled in the range of 1–3 eV, indicating the obvious contribution of the Cu‐O bond at the heterojunction interface to this interaction. Therefore, the catalytic activity of the CMCO nanozymes was greatly improved after the in situ oxidation of Cu_2_O to form the Mn_3_Cu_3_O_8_ shell. Because the catalytic reaction mainly occurs on the surface of the nanozymes, the d‐orbitals of the Mn_5_O_8_ shell and Mn_3_Cu_3_O_8_ shell were analyzed to compare the enzyme activities of CMO and CMCO, respectively. As shown in Figure 3G, the center of the d‐band of Mn_3_Cu_3_O_8_ is ≈2.32 eV, whereas that of Mn_5_O_8_ is ≈−2.93 eV. A portion of the Mn density of states is distributed around *E*_f_ = −6 eV, whereas the density of states for Cu is concentrated and close to the Fermi level, which makes the center of the d‐band of Mn_3_Cu_3_O_8_ closer to the Fermi level, thus causing Mn_3_Cu_3_O_8_ to be more active than Mn_5_O_8_. Thus, CMCO is more likely to undergo redox reactions than CMO. As shown in Figure S21 (Supporting Information), in the three enzyme‐catalyzed reactions, the GSHOx‐like and CAT‐like activities involved electron‐withdrawing reactions, whereas the POD‐like activity is associated with electron loss. The redox abilities of the CMO and CMCO nanozymes were calculated and analyzed by determining the electrical potential. As shown in Figure 3H, the electrostatic potential of CMCO was 6.7184 eV, which is higher than that of CMO (6.1874 eV); hence, the electron‐withdrawing ability of CMCO was stronger. Theoretically, CMCO should exhibit stronger GSHOx‐like and CAT‐like activities than CMO, whereas the POD‐like activity should be lower than that of CMO, which is consistent with previous measurements of the enzyme‐mimicking activity. Based on the above results, the enzyme‐mimicking characteristics of CMCO are more suitable for inducing cuproptosis compared to those of the other therapeutic agents; therefore, the CMCO nanozymes were used in the subsequent studies.

**Figure 4:**
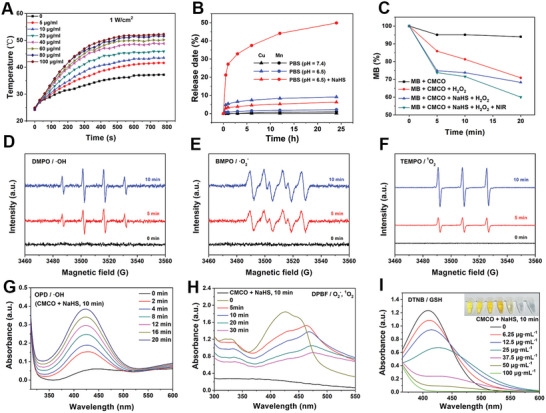
A) Heating curve of the products of different concentration CMCO reacting with NaHS (200 µg·mL^−1^) for 10 min under 1064 nm irradiation (1 W·cm^−2^). B) Cu and Mn ions released from CMCO in simulated normal tissue microenvironment (PBS, pH 7.4), simulated general TME solution (PBS, pH 6.5) and simulated colorectal TME solution (PBS, pH 6.5 containing NaHS). C) Degradation of MB due to the generation of ROS in the different groups (30 µg·mL^−1^ of MB, 40 µg·mL^−1^ of CMCO, 100 µg·mL^−1^ of NaHS, 1 mM of H_2_O_2_, 1 W·cm^−2^ of 1064 nm irradiation, reaction of CMCO with NaHS for 10 min). The ESR spectra of D) DMPO/·OH, E) BMPO/O_2_^−^, and F) TEMPO/^1^O_2_ for the products of CMCO (10 µg·mL^−1^) reacting with NaHS (100 µg·mL^−1^) for 10 min in simulated general TME solution (PBS, pH 6.5 containing H_2_O_2_) at different time. G) ·OH generation curves with OPD as a probe of the products of CMCO (10 µg·mL^−1^) reacting with NaHS (100 µg·mL^−1^) for 10 min in the presence of H_2_O_2_ (1 mM) at different time. H) O_2_^−^ and ^1^O_2_ generation curves with DPBF as a probe of the products of CMCO (10 µg·mL^−1^) reacting with NaHS (100 µg·mL^−1^) for 10 min in PBS (pH 6.5) at different time. I) GSH consumption curves with DTNB as a probe of the products of CMCO (10 µg·mL^−1^) reacting with NaHS (100 µg·mL^−1^). The products concentrations in illustration from left to right: 0, 6.25, 12.5, 25, 37.5, 50, 100 µg·mL^−1^.

To investigate the efficacy of the CMCO nanozymes for the colorectal TME‐activated bio‐reaction, PBS (pH 6.5) containing NaHS·xH_2_O was used to simulate the colorectal TME (weakly acidic with H_2_S overexpression). As shown in Figure S22 (Supporting Information), the weak absorbance of the CMCO nanozymes in the NIR‐II region gradually increased with the duration of sulfidation in the presence of NaHS. CMCO was reacted with NaHS for 5, 10, 20, and 60 min (Figure S23, Supporting Information). The photothermal conversion efficiency of the sulfurized products was 15.74%, 19.54%, 27.81%, and 39.20%, respectively. Nevertheless, limited by the complexity of the TME and insufficient reaction time, CMCO may not completely react with H_2_S during the initial stages of entering the colorectal tumor site. Therefore, the reaction time of CMCO with NaHS was shortened to simulate the incomplete sulfuration of CMCO in the TME. As shown in **Figures** **4**A, Figures S24 and S25 (Supporting Information), the products formed after 10 min of reaction between CMCO and NaHS (partial sulfidation) exhibited mild photothermal properties under 1064 nm laser irradiation. With an increase in the concentration of NaHS (from 0 to 100 µg·mL^−1^) and CMCO (from 0 to 200 µg·mL^−1^) or when the power density of the laser was increased (from 0.25 to 1.25 W·cm^−2^), the temperature generated by the reaction products under 1064 nm irradiation gradually increased. Excluding the absorption of water, pure CMCO had almost no photothermal effect except after the reaction with NaHS. The plot of the variation of the temperature when the NIR irradiation was turned on/off (Figure S26, Supporting Information) indicated good photothermal stability of the partially sulfurized CMCO. The thermal images (Figure S27, Supporting Information) show that the temperature of partially sulfurized CMCO increased rapidly from 24.3 °C (room temperature) to 40.0 °C at 4 min upon 1064 nm laser irradiation, and reached 51.8 °C at 10 min. These results suggest that the CMCO nanozymes should display excellent photothermal properties after partial activation by H_2_S in the colorectal TME, which can prevent photothermal damage to normal tissues. The Cu and Mn ions released from CMCO in different simulated microenvironment solutions were detected using inductively coupled plasma mass spectrometry (ICP‐MS, Figure 4B). The release of Cu ions from CMCO in PBS at pH 6.5 was higher than that in PBS at pH 7.4, whereas the release of Cu ions was slightly reduced owing to partial sulfidation in PBS (pH 6.5, containing NaHS), resulting in cuproptosis. A massive amount of Mn ions was released in PBS at pH 6.5 containing NaHS compared to that in PBS at pH 6.5 and pH 7.4 (Figure 4B), indicating that sulfidation on the surface of CMCO accelerated the release of Mn ions to induce ferroptosis. Maintaining structural stability in the colorectal TME is essential for expressing the enzymatic activity of the CMCO nanozymes. As shown in Figure S28A (Supporting Information), in PBS at pH 6.5 with NaHS, the core‐shell structure of the CMCO nanozymes was retained for 10 min, and the size was similar to that of the original CMCO (Figure S29, Supporting Information). More importantly, there were no other crystal peaks in the XRD pattern of CMCO that was sulfurized for 10 min (Figure S28B, Supporting Information), and Cu and Mn were still in the Cu^+/2+^and Mn^2+/3+/4+^ valence states (Figure S30, Supporting Information), which made it possible for the partially sulfurized CMCO to exhibit multiple enzyme activities. Methylene blue (MB) served as a ROS indicator for evaluating the catalytic activity and photothermal effect of CMCO after sulfuration with NaHS. As shown in Figure 4C and Figure S31 (Supporting Information), the intensity of the characteristic absorption peak of MB decreased significantly over time in the presence of H_2_O_2_, indicating that a large amount of ROS was generated and participated in MB degradation. Thus, CMCO partially sulfurized with NaHS retained its ability to generate ROS. The ROS generation ability was further enhanced under the photothermal effect induced by 1064 nm laser irradiation, which indicates that the photothermal effect could enhance the catalytic activity of partially sulfurized CMCO nanozymes. Electron spin resonance (ESR) spectra were acquired to detect the free radicals generated by the partially sulfurized CMCO and original CMCO nanozymes. 5,5‐Dimethyl‐1‐pyrroline‐*N*‐oxide (DMPO), as a capture agent, was used to detect the generation of hydroxyl radicals (·OH) (Figure 4D; Figure S32A, Supporting Information); 3,4‐dihydro‐2‐methyl‐1,1‐dimethylethyl ester‐2H‐pyrrole‐2‐carboxylic acid‐1‐oxide (BMPO) was used for superoxide anions (O_2_^−^) (Figure 4E; Figure S32B, Supporting Information) and 2,2,6,6‐tetramethyl‐1‐piperidinyloxy (TEMPO) for singlet oxygen (^1^O_2_) (Figure 4F; Figure S32C, Supporting Information). The results showed that the CMCO nanozymes could catalyze the generation of ·OH, O_2_^−^, and ^1^O_2_ both before and after partial sulfurization in TME. Furthermore, the enzymatic activity of the CMCO nanozymes after partial sulfurization by NaHS was examined. As shown in Figure 4G,H, the partially sulfurized CMCO nanozymes could also catalyze the generation of ·OH, O_2_^−^, and ^1^O_2_. The ability of the partially sulfurized CMCO nanozymes to catalyze the consumption of GSH and the production of O_2_ from H_2_O_2_ was examined (Figure 4I; Figure S33, Supporting Information). Overall, the CMCO nanozymes possess colorectal TME‐responsive properties that induce a photothermal effect that enhances the enzyme‐mimicking activity.

**Figure 5:**
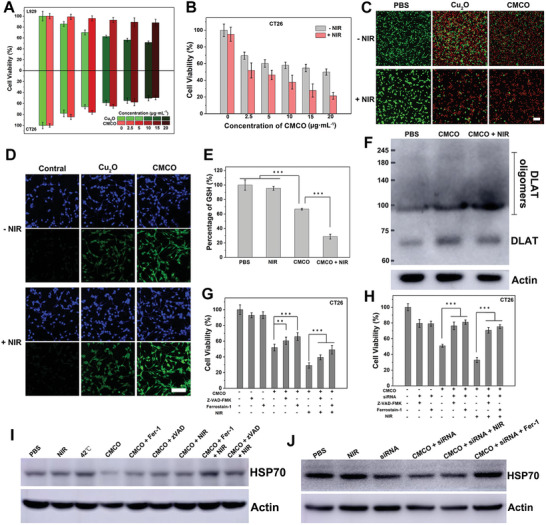
A) Cytotoxicity assessment on L929 and CT26 cells treated with different concentration of Cu_2_O and CMCO nanozymes. Dates are presented (*n* = 6). B) Cytotoxicity assessment on CT26 cells treated with different concentration of CMCO nanozymes with or without 1064 nm laser irradiation. Dates are presented (*n* = 6). C) The live/dead staining assay on CT26 cells after different treatments (scale bar: 50 µm). D) The detection of intracellular ROS after different treatments on CT26 cells (scale bar: 50 µm). E) The contents of intracellular of GSH on CT26 with different treatments. Dates are presented (*n* = 3). ^***^ *p* < 0.001 by two‐tailed Student's *t*‐test. F) Western blot of DLAT and DLAT oligomers after different treatments. G) and H) Cytotoxicity assessment on CT26 cells with different treatments. Dates are presented (*n* = 6). ^**^ *p* < 0.01 and ^***^ *p* < 0.001 by two‐tailed Student's *t*‐test. I,J) Western blot of HSP70 after different treatments to investigate that CMCO nanozymes induced Ferroptosis to consume HSP70 to achieve mild‐phototherapy and enhance cuproptosis. For NIR irradiation, 1064 nm laser (1 W·cm^−2^, 5 min) was used and the temperature was controlled within 42 °C.

Inspired by the excellent performance of the CMCO nanozymes, the ferroptosis‐boo sted cuproptosis induced by a mild photothermal effect was investigated in vitro. Before the cell experiments, all nanoparticles were surface‐modified with bovine serum albumin (BSA), which is typically used to improve the biocompatibility of materials.^[^ ^26^ ^]^ As shown in Figures S34, S35, and S36 (Supporting Information), the BSA coating did not affect the structure, stability, enzyme activity, or photothermal properties of the CMCO nanozymes. First, the cytotoxicity of the CMCO nanozymes was evaluated in fibroblast L929 and colorectal tumor cells CT26 by methyl thiazolyl tetrazolium (MTT) assay. Cu_2_O nanoparticles were used as control materials. As shown in **Figure** **5**A, the CMCO nanozymes exhibited favorable biocompatibility with L929 cells, whereas the cells that were directly exposed to Cu_2_O experienced a significant toxic effect. These results indicate that the formation of the Mn_3_Cu_3_O_8_ shell on the surface of Cu_2_O prevented direct exposure of the cells to the Cu_2_O core, thereby reducing the cytotoxicity to normal cells. To further test this hypothesis, normal mouse colonic smooth muscle cells (CSMC) were treated with different concentrations of CMCO or Cu_2_O. As shown in Figure S37 (Supporting Information), the cell viability of the CMC‐treated groups was higher than that of the groups treated with Cu_2_O at various concentrations. However, the cytotoxicity of the CMCO nanozymes to CT26 cells was higher than the cytotoxicity to L929 cells. The CMCO nanozymes were more toxic to CT26 cells than Cu_2_O when the concentration of the nanoparticles was increased to 20 µg·mL^−1^. The CMCO nanozymes were effective against tumor cells in other cell lines, including murine and human cell lines (Figures S38 and S39, Supporting Information). The microenvironment of tumor cells differs from that of normal cells. The CMCO nanozymes exhibit responsiveness to the colorectal TME; thus, the outstanding catalytic activity of the CMCO nanozymes in CT26 cells was the key factor inducing tumor cell death. As shown in Figure 5B, the killing rate of CMCO for CT26 cells was enhanced under 1064 nm laser irradiation. To illustrate this effect, during the experiment, the temperature was measured by constructing a thermograph and was controlled within 42 °C under NIR irradiation. The results show that the mild photothermal effect could improve the therapeutic effect of the CMCO nanozymes through enhanced enzyme‐mimicking activity in colorectal cancer cells. Similar results were observed in the live/dead cell staining images (Figure 5C), where few living cells were observed in the CMCO + NIR group. Intracellular ROS generation was detected using a ROS assay kit. As shown in Figure 5D, a large amount of ROS was generated in the CMCO + NIR group due to the photothermal effect, which enhanced the enzyme‐mimicking activity of the CMCO nanozymes. ROS generation favors cuproptosis. CMCO releases Cu and Mn ions into the colorectal TME. The effects of these ions are similar to those of Fe ions in inducing ferroptosis.^[^ ^5^ ^]^ As shown in Figure 5E, the intracellular GSH content decreased after the cells were treated with the CMCO nanozymes, especially after NIR irradiation, which further promoted ferroptosis. The accumulation of LPO from iron metabolism is an important marker of ferroptosis; therefore, the intracellular expression of LPO was evaluated to confirm the occurrence of ferroptosis in CMCO. As shown in Figure S40 (Supporting Information), significant accumulation of LPO was detected in the CMCO group, and the fluorescence signal increased after NIR irradiation, indicating that ferroptosis was induced by CMCO and was further enhanced by the mild photothermal effects. Moreover, the free Cu^2+^ and Cu^+^ both decreased with increasing amounts of GSH, indicating that intracellular GSH can chelate the Cu ions released from CMCO, thereby inhibiting cuproptosis (Figure S41, Supporting Information). Therefore, GSH depletion by GSHOx‐like of CMCO prevents the chelation of Cu ions, thus inducing cuproptosis. Theoretically, we can achieve enhanced coproptosis at the cellular level. According to an original report by Tsvetkov, oligomerization of the DLAT enzyme is an important marker of cuproptosis. As shown in Figure 5F, treatment with CMCO resulted in obvious oligomerization of lipoylated DLAT, and more oligomers were detected under NIR irradiation, indicating that the mild photothermal effect could enhance carboxyproptosis. Subsequently, the occurrence of coproptosis was verified. FDX‐1 is a key regulatory factor in coproptosis. A silencer (FDX‐1 siRNA) was used to silence the FDX‐1 gene and inhibit protein expression to prevent carcinogenesis. As shown in Figure S42 (Supporting Information), the cell activity improved after the addition of FDX‐1 siRNA. The expression of FDX‐1 proteins in each group was detected using western blotting (WB). As shown in Figure S43 (Supporting Information), the highest expression of FDX‐1 proteins was observed in the CMCO group, indicating that the CMCO nanozymes induced cuproptosis, which requires a large number of FDX‐1 proteins for regulation. The expression of the FDX1 protein was reduced after the addition of FDX‐1 siRNA, which could result in the inhibition of cuproptosis.

To investigate a possible mechanism (including apoptosis, ferroptosis, and cuproptosis) for the induction of cell death via the mild photothermal effect, Z‐VAD‐FMK (zVAD, apoptosis inhibitor), Ferrostain‐1 (Fer‐1, ferroptosis inhibitor), and FDX‐1 siRNA were used in the cell viability assessment experiments. Notably, as an artificial synthetic antioxidant, Ferrostain‐1 achieves the goal of inhibiting ferroptosis via a reducing mechanism, thereby preventing damage to membrane lipids and thus suppressing apoptosis by depleting intracellular ROS. As shown in Figure 5G and Table S2 (Supporting Information), CT26 cell death was induced by apoptosis (8.6%), ferroptosis (5.3%), and cuproptosis (34.2%) without NIR irradiation. CT26 cell death was induced by apoptosis (10.3%), ferroptosis (9.7%), and cuproptosis (50.9%) under NIR irradiation. The increased effect of the three cell death mechanisms, especially cuproptosis, was caused by the mild photothermal effect, which enhanced the enzyme‐mimicking activity of the CMCO nanozymes. As shown in Figure 5H, FDX‐1 siRNA was used to inhibit cuproptosis. The cell viability in the CMCO + siRNA + Z‐VAD‐FMK and CMCO + siRNA + Ferrostain‐1 system returned to approximately the level of the control groups (siRNA + Z‐VAD‐FMK and siRNA + Ferrostain‐1). Similarly, the cell viability was restored by NIR irradiation. These results indicate that the toxic effects of CMCO nanozymes on colorectal cancer cells were mainly caused by cuproptosis. GPX4 inactivation is another manifestation of ferroptosis. CMCO down‐regulated the expression of GPX4 (Figure S44, Supporting Information), confirming the occurrence of ferroptosis.

To investigate the mechanism of cuproptosis, the expression of HSP70 protein was measured by WB. Both heat and coproptosis can induce the expression of HSP70 proteins, thereby inhibiting the killing effect on tumor cells. As shown in Figure 5I, HSP70 was highly expressed at 42 °C, but decreased after treatment with CMCO nanozymes. Moreover, treatment with Ferrostain‐1 (group of CMCO + Fer‐1 + NIR) restored the high expression of HSP70, whereas Z‐VAD‐FMK (CMCO + zVAD + NIR) did not. These results indicate that HSP70 downregulation is directly regulated by ferroptosis, but not by apoptosis. As shown in Figure 5J, the expression of HSP70 was down‐regulated in the CMCO + siRNA and CMCO + siRNA + NIR groups during ferroptosis. Importantly, the expression of HSP70 was significantly restored after treatment with Ferrostain‐1, thereby inhibiting ferroptosis (CMCO + siRNA + Fer‐1 group), which demonstrates that ferroptosis can boost cuproptosis by downregulating HSP70. During cellular respiration, oxygen is the final electron receptor in the electron transfer chain (ETC) and is involved in water generation. Therefore, increasing the O_2_ content in tumor cells results in more electron receptors in the ETC, which facilitates the regeneration of NADH^+^ and FAD to promote the TCA cycle.^[^ ^27^ ^]^ In a study by Tsvetkov, ECT was found to be essential for cuproptosis, and O_2_ was a factor that promoted cuproptosis.^[^ ^3^ ^]^ Overall, the TCA cycle indirectly requires O_2_ participation. Interestingly, in a recent study, Zhang et al. explained the role of O_2_ in cuproptosis from a different perspective. In their study, (BRD4) inhibitor JQ‐1 inhibited glycolysis in combination with oxygen, leading to a reduction in ATP, which blocked ATP7B (major export proteins that depend on ATP for energy and control copper efflux) and further increased intracellular copper accumulation, thereby enhancing cuproptosis.^[^ ^28^ ^]^ As shown in Figure S45 (Supporting Information), the viability of CT26 cells treated with CMCO under normoxic conditions (21% O_2_) was significantly lower than that under hypoxic conditions (1% O_2_). In the present study, due to its CAT‐like activity, the CMCO nanozyme reacted with endogenous H_2_O_2_ to generate O_2_, reversing the hypoxic microenvironment, thereby increasing cuproptosis. Therefore, due to its CAT‐like activity, the CMCO nanozyme catalyzes the overexpression of H_2_O_2_ in the TME to generate O_2_ and promotes cuproptosis by enhancing mitochondrial respiration and inhibiting Cu^+^ efflux. As shown in Figure S46 (Supporting Information), the POD‐like, GSHOx‐like, and CAT‐like activities of CMCO were all enhanced with increasing temperature, which indicates that the photothermal effect of partially sulfurized CMCO in the colorectal TME under NIR irradiation could improve the enzyme‐mimicking activity, thereby increasing the therapeutic effect. Based on the above analysis of CT26 cell death induced by CMCO nanozymes via various processes, a mechanism was proposed to explain the interplay among the enzyme mimicking activity, mild photothermal effect, ferroptosis, and cuproptosis (Figure S47, Supporting Information). The GSHOx‐like activity of the CMCO nanozymes depleted GSH to promote cuproptosis and ferroptosis. The CAT‐like activity catalyzes the production of O_2_, which contributes to enhancing cuproptosis. Stress‐expressed HSPs were consumed by ROS produced by the OXD‐like and POD‐like enzyme‐mimicking activity of the nanozymes, and LPO was accumulated through ferroptosis, thus mitigating the inhibition of ferroptosis. More importantly, the mild photothermal effect enhanced the enzyme‐mimicking activity of the CMCO nanozymes, which further promoted the biochemical reaction processes described above. In summary, we achieved high‐efficiency ferroptosis‐boosted‐cuproptosis induced by a mild photothermal effect by using colorectal TME‐responsive CMCO nanozymes.

**Figure 6:**
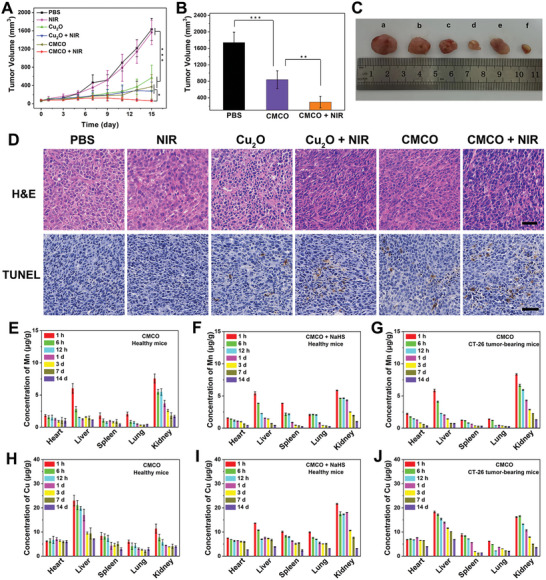
A) The tumor growth curves of CT26 tumor‐bearing mice after different treatments with intratumor injection. Dates are presented (*n* = 6). ^*^ *p* < 0.05, ^***^ *p* < 0.001 by one‐way ANOVA with Bonferroni post‐hoc test. B) The tumor volumes of CT26 tumor‐bearing mice after different treatment with intravenous injection. Dates are presented (*n* = 6). ^**^ *p* < 0.01, ^***^ *p* < 0.001 by one‐way ANOVA with Bonferroni post‐hoc test. C) Photographs of excised tumor after treated with a) PBS, b) NIR, c) Cu_2_O, d) Cu_2_O + NIR, e) CMCO, and f) CMCO + NIR via intratumor injection. D) H&E and TUNEL staining of tumor tissues after different treatments (scale bars: 100 µm). E–J) The concentration of Mn or Cu in major organs after treatments with intravenous injection at different time point. E,H) CMCO and F,I) CMCO + NaHS for healthy mice. G,J) CMCO for CT26 tumor‐bearing mice. Dates in (E–J) are presented as mean ± SD (*n* = 3).

After confirming the anticancer effects of CMCO on colorectal cancer at the cellular level, we investigated its therapeutic effect in vivo. Briefly, BALB/c mice bearing CT26 tumors were subjected to different treatments (PBS, NIR, Cu_2_O, Cu_2_O + NIR, CMCO, and CMCO + NIR), and the anticancer efficacy was assessed. For the groups exposed to irradiation, a 1064 nm laser (1 W·cm^−2^) was used to irradiate the tumor site for 10 min (irradiation for 5 min, interruption for 5 min, and irradiation for another 5 min) 10 min after injection of the drug; the temperature of the tumors was controlled within 42 °C. As shown in **Figure** **6**A and Figure S48 (Supporting Information), when the mice were treated by intratumoral injection, obvious tumor growth suppression was observed in the groups treated with Cu_2_O and CMCO compared to that in the groups treated with PBS and NIR. Moreover, tumor growth was inhibited by NIR irradiation. Photographs of the excised tumors of the different groups showed the same results (Figure 6C). The body weight of the mice in the different treatment groups remained stable (Figure S49, Supporting Information). CMCO nanozymes were intravenously injected to investigate their therapeutic effects based on the enhanced permeability and retention (EPR) effect (Figure S50, Supporting Information). The final tumor volume, tumor growth curves, and weights of the mice after intravenous injection are shown in Figure 6B and Figure S51 (Supporting Information). Tumor growth in the CMCO group was significantly inhibited compared with that in the PBS group. Furthermore, comparison of the CMCO and CMCO + NIR groups showed that mild photothermal treatment enhanced tumor suppression. After therapy, histological analysis of the main organs (heart, liver, spleen, lung, and kidney) revealed no obvious abnormalities or damage, indicating the safety of the therapeutic regimen based on CMCO nanozymes (Figure S52, Supporting Information). Hematoxylin and eosin (H&E) staining and terminal deoxynucleotidyl transferase dUTP nick labeling (TUNEL) were used to assess the excised tumors, which indicated that the CMCO + NIR group had the most severe damage (Figure 6D). These results suggest that CMCO is prospectively effective in the treatment of colorectal cancer under 1064 nm irradiation.

The CMCO nanozymes were expected to enter the tumor and react with overexpressed H_2_S to generate ultrasmall nanoparticles, which could be excreted through the kidney, avoiding the long‐term toxicity of inorganic nanoparticles to the body. Thus, the colorectal TME‐activated bio‐decomposition of the CMCO nanozymes was investigated. The TEM images (Figure S53, Supporting Information) and size distribution (Figure S54, Supporting Information) of CMCO in the presence of NaHS in PBS (pH 6.5) and PBS (pH 7.4) indicate that a weakly acidic environment could expedite dissolution of the Mn_3_Cu_3_O_8_ shell and stimulate the reaction of Cu_2_O with overexpressed H_2_S in the colorectal TME. As shown in Figure S55 (Supporting Information), Cu_2_O reacted rapidly (within 1 min) with NaHS to generate ultrasmall nanoparticles, where the reaction product was Cu_7_S_4_ (PDF#23‐0958). As shown in Figure S56 (Supporting Information), CMCO also completely dissolved into ultra‐small nanoparticles after reaction with NaHS for 1 h in PBS (pH 6.5). The biodistribution of Mn and Cu ions in the major organs was examined using ICP‐MS. As shown in Figure 6E,F,G, the Mn ions in the different treatment groups were mainly metabolized through the kidney, which suggests that the Mn_3_Cu_3_O_8_ shell was easily decomposed into ultrasmall Cu_7_S_4_ nanoparticles after being injected into the body. These results correspond to the process used to form the Mn_3_Cu_3_O_8_ shell for transient protection of normal cells from direct exposure to the Cu_2_O core. As shown in Figure 6H, Cu ions were mainly metabolized in the liver when CMCO was injected into healthy mice through the tail vein, which may be due to the large size of Cu_2_O. However, when the products of the reaction between CMCO and NaHS were injected into healthy mice, Cu ions were primarily excreted from the kidneys (Figure 6I). CMCO that was injected into CT26 tumor‐bearing mice was partially sulfurized and decomposed in the colorectal TME, and was mainly excreted through the kidney and liver (Figure 6J). Moreover, Figure S57 (Supporting Information) shows that Mn and Cu were almost completely metabolized from the body through the feces and urine. Finally, the biochemical parameters of the liver and kidney in all treatment groups were within normal ranges (Figure S58, Supporting Information). In general, CMCO nanozymes not only efficiently inhibited colorectal cancer, but also exhibited excellent biosecurity in vivo.

## 3 Conclusion

In summary, a core‐shell‐structured Cu_2_O@ Mn_3_Cu_3_O_8_ (CMCO) nanozyme capable of inducing high efficiency ferroptosis‐boosted‐cuproptosis via mild‐photothermal effect for colorectal cancer therapy was synthesized. Due to the presence of Cu^+^/Cu^2+^ and Mn^2+^/Mn^3+^/Mn^4+^ redox couple, CMCO nanozyme exhibited a variety of enzyme‐mimicking (GSHOx‐like, POD‐like, CAT‐like) activity. CMCO exhibits stronger catalytic activity than Cu_2_O and analogue Cu_2_O@Mn_5_O_8_ (CMO) owing to the better band continuity as conductive material and closer to the Fermi level. CMCO nanozymes were partially sulfurized by specifically overexpressed H_2_S in the simulated colorectal TME, which not only released Cu and Mn ions, but also presented stable photothermal property, while maintaining the enzyme‐mimicking activity. The cellular level studies suggest that CMCO nanozymes showed good biocompatibility for normal cells due to the protective of Mn_3_Cu_3_O_8_ shell, but enormous toxic for colorectal cells, especially under the mild‐photothermal effect. The stress‐expressed HSPs were down‐regulated by enhanced ferroptosis induced by CMCO nanozymes, thus further sensitizing cuproptosis. Moreover, CMCO nanozymes exhibited prominent tumor growth inhibition performance and biosecurity in vivo. Collectively, this work first reports mild‐photothermal effect induce high efficiency ferroptosis‐boosted‐cuproptosis based on nanozyme, which provides a safe and feasible strategy for the treatment of colorectal cancer.

## 4 Experimental Section

### Materials

All reagents were used directly without further purification. Copper (II) nitrate trihydrate (Cu(NO_3_)_3_·3H_2_O), hydrazine hydrate (N_2_H_4_·H_2_O), Polyvinylpyrrolidone (PVP, *M*_W_ = 58 000), *O*‐Phenylenediamine (OPD), methylene blue (MB), 5,5′‐Dithiobis‐(2‐nitrobenzoic acid) (DTNB), 1, 3‐diphenylisobenzofuran (DPBF) and 3‐(4,5‐dimethylthiazole)−2,5‐diphenyltetrazolium bromide (MTT) were purchased from Aladdin (Shanghai, China). Sodium hydrosulfide hydrate (NaSH·xH_2_O), Hydrogen Peroxide (H_2_O_2_), and Glutathione (reduced) were purchased from Macklin (Shanghai, China). Potassium permanganate (KMnO_4_) was purchased from Sinopharm (Beijing, China). Bovine serum albumin (BSA) was purchased from BioFroxx (Guangzhou, China). Bradford Protein Assay Kit, Hoechst, Reactive Oxygen Species (ROS) Assay Kit, and Calcein‐AM/PI Double Stain Kit were purchased from Beyotime (Shanghai, China). Reduced glutathione (GSH) assay kit was purchased from Solarbio (Beijing, China). BCA protein assay kit, RPMI‐1640 medium, dulbecco's modified eagle medium (DMEM), fetal bovine serum (FBS), penicillin‐streptomycin and enhanced chemiluminescence (ECL) substrate kit were purchased from ThermoFisher Scientific (Shanghai, China). FDX‐1 siRNA and Transfection Reagent were purchased from RIBOBIO (Guangzhou, China). Ferrostain‐1 was purchased from Sigma‐Aldrich (Shanghai, China). Z‐VAD‐FMK was purchased from TargetMol (Shanghai, China).

### Apparatus

The Transmission electron microscopy (SEM) images were obtained using a Talos F200x SEM (ThermoFisher, USA). The X‐ray diffraction (XRD) patterns were recorded on an Empyrean X‐ray powder diffractometer (PANalytical, Netherlands). The X‐ray photoelectron spectra (XPS) patterns were recorded on an Escalab Xi electron spectrometer (ThermoFisher, USA). The dynamic light scattering (DLS) was measured on the NANO ZS Malvern instrument (UK). The hydroxyl radical (·OH), superoxide anion (O_2_^−^), and singlet oxygen (^1^O_2_) were verified by an A300 electron spin resonance (ESR) spectrometer (Bruker, Germany). The UV–vis‐NIR adsorption spectrum was measured on a UV3600 spectrophotometer (Shimadzu, Japan). The ions of Cu and Mn were detected on the iCAP RQ Inductively Coupled Plasma‐mass spectrometry (ICP‐MS) instrument (ThermoFisher, USA). The thermal images were recorded using a T420 thermal camera (FLIR, USA).

### Synthesis of Cu_2_O

3.5 g PVP was scattered into 50 mL Cu(NO_3_)_3_·3H_2_O (0.02 M) under drastic magnetic stirring for 30 min. And then, 552 µL N_2_H_4_·H_2_O (35 wt.%) was added into the above mixtures. After 1 h, Cu_2_O was obtained by alternating centrifugation with deionized water and ethanol. At last, Cu_2_O was dispersed in 5 mL ethanol for storing.

### Synthesis of Cu_2_O@Mn_3_Cu_3_O_8_ (CMCO)

KMnO_4_ (20 mg) was dissolved in 20 mL deionized water, and then 1 mL Cu_2_O was added in it. After magnetic stirring for 2 h, Cu_2_O@Mn_3_Cu_3_O_8_ (CMCO) was obtained by centrifugation with water, and dispersed in 5 mL water for storing. 50 mg BSA was added into CMCO under magnetic stirring for 12 h to be applied in cell and animal experiment.

### Synthesis of Cu_2_O@Mn_5_O_8_ (CMO)

1 mL Cu_2_O was centrifugated and dispersed in 20 mL deionized water. 50 mg BSA was dissolved in Cu_2_O aqueous solution and magnetic stirred for 6 h. Cu_2_O@ BSA was obtained by centrifugation, and dispersed in 10 mL water. 10 mg KMnO_4_ was dissolved in 10 mL deionized water and dropwise added into Cu_2_O@BSA aqueous solution. After magnetic stirring for 1 h, Cu_2_O@Mn_5_O_8_ (CMO) was obtained by centrifugation with water, and dispersed in 5 mL water for storing.

### Synthesis of BSA‐Coated CMCO and BSA‐Coated Cu_2_O

The surface electrostatic adsorption method was used to obtain BSA‐coated CMCO and BSA‐coated Cu_2_O for in vitro and in vivo experiments. Specifically, the mixed solution composed of CMCO or Cu_2_O and BSA at a concentration ratio of 1:2 was stirred for 4 h, and the BSA‐coated CMCO or Cu_2_O was obtained after centrifugation and washing.

### Cell Culture

CT26 and L929 cells were cultured in RPMI 1640 medium supplemented with 1% (v/v) penicillin/streptomycin, and 10% (v/v) fetal bovine serum (FBS) at 37 °C under 5% CO_2_. The medium was replaced every other day.

### Establishment of Animal Model

Male Balb/c mice (six weeks) were bought from Vital River laboratories (Guangzhou, China), and housed in specific‐pathogen‐free facilities in Laboratory Animal Center of Guangzhou Medical University. All the animal studies were performed in accordance with the regulations of Laboratory Animal Ethics Committee of Guangzhou Medical University (ethics approval number: GY2022037). The CT26 tumor‐bearing mice were established as the animal model by subcutaneously injecting CT26 cells (3 × 10^6^ cells, 100 µL) into the right dorsal region of mice. The tumor grew to 100 mm^3^ for the next experiment.

### Statistical Analysis

The number of samples (n) for each statistic is indicated in the relevant figure legends. Dates were expressed as mean ± standard deviation (SD). SPSS 18.0 software was used to process the measurement data. Student's *t*‐test was used for two‐group comparisons in relevant cell experiment, and one‐way analysis of variance (ANOVA) with Bonferroni post‐hoc test was used for multi‐group comparisons in relevant animal experiment. In this paper, the differences were considered to be statistically significant when *p* < 0.05 (^*^).

More experimental methods are available in the Supporting Information.

## Conflict of Interest

The authors declare no conflict of interest.

## Supporting information

Supporting Information

## Data Availability

The data that support the findings of this study are available from the corresponding author upon reasonable request.
