# Supplementary material for: Mild‐Photothermal Effect Induced High Efficiency Ferroptosis‐Boosted‐Cuproptosis Based on Cu2O@Mn3Cu3O8 Nanozyme
Source: Adv Sci (Weinh). 2023 Oct 11;10(33):2303694. doi: 10.1002/advs.202303694 (PMC10667815; doi:10.1002/advs.202303694)
Supplement: Supplementary file 1 — Supporting Information [file ADVS-10-2303694-s001.pdf]

ADVANCED SCIENCE
Open Access

Supporting Information

for *Adv. Sci.*, DOI 10.1002/advs.202303694

Mild-Photothermal Effect Induced High Efficiency Ferroptosis-Boosted-Cuproptosis Based on $Cu_2O@Mn_3Cu_3O_8$ Nanozyme

*Wei Chen, Wenyu Xie, Zhimin Gao, Chen Lin, Meiling Tan, Yaru Zhang and Zhiyao Hou**

Supporting Information

Mild-Photothermal Effect Induced High Efficiency Ferroptosis-Boosted-Cuproptosis Based on $Cu_2O@Mn_3Cu_3O_8$ Nanozyme

*Wei Chen, Wenyu Xie, Zhimin Gao, Chen Lin, Meiling Tan, Yaru Zhang, Zhiyao Hou**

Experimental Section

Weakly acid-responsive ions release

CMCO was dissolved in PBS (pH= 7.4), PBS (pH= 6.5) and PBS (pH = 6.5) + NaHS, and put in a shaking incubator (60 rpm, 37 °C). Then, the supernatant solution was collected by centrifugation at different time-point. The Cu and Mn ions release concentration were measured by inductively coupled plasma mass spectroscopy (ICP-MS).

Vis-NIR absorption of CMCO reacting with $NaHS{\cdot}xH_2O$

NaHS (200 μg·mL^{-1}) was added into CMCO (100 μg·mL^{-1}) solution, and the absorption value at 600-1200 nm was detected by UV-vis-NIR spectrometer at 5, 10, 20, 40, 60 min, respectively.

Measurements of mimic enzyme activity assays

The POD-like activity kinetic assays of Cu_2O, CMO and CMCO with H_2O_2 as the substrate were performed. Cu_2O/CMO/CMCO (20 μg·mL^{-1}), OPD (1 mM) and different concentrations (0.5, 1, 2.5, 5, 10, 20, 30 mM) of H_2O_2 were added into PBS (pH = 6.5) for reacting 300 s. For each H_2O_2 concentration, the initial reaction rates (V_0) were calculated from absorbance changes at 420 nm by Beer-Lambert Law (equation 1, ε: 17200 M^{-1}·cm^{-1} of oxOPD).

$$A = \varepsilon bc \qquad \text{equation 1}$$

The GSHOx-like activity kinetic assays of Cu_2O, CMO and CMCO with GSH as the substrate were performed. The supernatant solution collected from centrifugation of Cu_2O/CMO/CMCO (50 μg·mL^{-1}) and different concentrations (0.2, 0.4, 0.6, 0.8, 1, 1.2 mM) of GSH for reacting 30 s, was added into PBS (pH = 6.5) containing DTNB (100 μg·mL^{-1}) for reacting 10 min. For each GSH concentration, the actually GSH reacting concentrations by Cu_2O/CMO/CMCO were decided from absorbance changes at 410 nm by Beer-Lambert Law (equation 1, ε: 13600 M^{-1}·cm^{-1} of TNB), and then the initial reaction rates (V_0) were calculated.

The CAT-like activity kinetic assays of Cu_2O, CMO and CMCO with H_2O_2 as the substrate were performed. Cu_2O/CMO/CMCO (50 μg·mL^{-1}) and different concentrations (2, 4, 8, 12, 16, 20 mM) of H_2O_2 were added into PBS (pH = 6.5) for reacting 30 s. The real-time oxygen concentration was recorded using portable dissolved oxygen meter. For each H_2O_2 concentration, the initial reaction rates (V_0) were decided from the real-time oxygen concentration changes.

The values of K_m and V_{max} can be calculated according to the initial reaction rates (V_0) with different initial substrate ($[S]$) by Lineweaver-Bruke plot (equation 2).

$$\frac{1}{V_0} = \frac{K_m+[S]}{V_{max}[S]} = \frac{K_m}{V_{max}}\frac{1}{[S]} + \frac{1}{V_{max}} \quad \text{equation 2}$$

Theoretical calculation

The calculations were based on density functional theory (DFT) using projector augmented wave (PAW) methods, as implemented in the Vienna ab initial simulation package (VASP). A plane-wave basis set with a kinetic-energy cut-off of 400 eV was used to expand the wave function of valence electrons. The generalized gradient approximation (GGA) with the Perdew-Burke-Ernzerhof (PBE) functional was used for describing the exchange-correlation interactions. The structural relaxations were performed by computing the Hellmann–Feynman forces within the total energy and force convergences of 10^{-5} eV and 10^{-4} eV/Å, respectively.

Photothermal conversion efficiency of CMCO and Cu_2O in the presence of NaHS

CMCO (100 μg·mL^{-1}) reacted with NaHS·xH_2O (200 μg·mL^{-1}) for 5, 10, 20, 60 min, respectively, and the products were dispersed in the same volume of deionized water after centrifugation. The aqueous solution (1 mL) was irradiated upon 1064 nm laser (1 W·cm^{-2}) for 780 s, and then turned off. In the meantime, the temperature was detected by infrared camera every 30 s.

The photothermal conversion efficiency (η) can be calculated according to the equation 3:

$$\eta = \frac{hS(T_{max}-T_{surr})-Q_{dis}}{I(1-10^{-A_{1064}})} \quad \text{equation 3}$$

Where h is the heat transfer coefficient, S is the surface area of the container. Here, hS can be obtained by the equation 4. T_{max} is the maximum temperature of the solution, and T_{surr} is the temperature of the surrounding. Q_{dis} is the heat generated after water and container absorbs light, which is calculated by the equation 5. I is the laser power density, and A_{1064} is the absorption value of the material at 1064 nm.

Photothermal effect of CMCO in the presence of NaHS

CMCO (100 μg·mL^{-1}) reacted with different concentration (0, 25, 50, 75, 100, 150, 200 μg·mL^{-1}) of NaHS·xH_2O for 10 min, and the products were irradiated upon 1064 nm laser (1 W·cm^{-2}). Different concentration (0, 5, 10, 20, 40, 60, 80, 100 μg·mL^{-1}) of CMCO reacted with NaHS·xH_2O (200 μg·mL^{-1}) for 10 min, and the products were irradiated upon 1064 nm laser (1 W·cm^{-2}). CMCO (100 μg·mL^{-1}) reacted with NaHS·xH_2O (200 μg·mL^{-1}) for 10 min, and the products were irradiated upon 1064 nm laser by different power density (0.25, 0.5, 0.75, 1, 1.25 W·cm^{-2}). In the meantime, the temperature was detected by infrared camera every 30 s.

To test the photothermal stability of CMCO in the presence of NaHS, the 1064 nm laser (1 W·cm^{-2}) was turned on for 600 s and turned off for 600s to the products, which were reacted by CMCO (100 μg·mL^{-1}) and NaHS·xH_2O (200 μg·mL^{-1}) for 10 min. Three cycles were repeated.

$$\tau_s = \frac{m_D C_D}{hS} \qquad \text{equation 4}$$

$$Q_{dis} = hS(T_{max,H_2O} - T_{surr}) \qquad \text{equation 5}$$

mD is the mass of water, and CD is the heat capacity of water (4.2 J·g^{-1}·°C^{-1}). τs is the sample system time constant, which was calculated using the equation 6 and equation 7.

$$t = -\tau_s ln\theta \qquad \text{equation 6}$$

$$\theta = \frac{T_{surr} - T}{T_{surr} - T_{max}} \qquad \text{equation 7}$$

t is the time of the cooling process after irradiation, and T is the temperature of the solution at different time point during this process.

Detection of free radical (·OH, $^1O_2$ and O_2^-)

The types of free radical produced by the products of CMCO (10 μg·mL^{-1}) reacting with NaHS (100 μg·mL^{-1}) for 10 min were detected via electron spin resonance (ERS) spectrometer. 5,5-dimethyl-1-pyrroline-N-oxide (DMPO) as capture agent was used to detect the generation of ·OH, 3,4-dihydro-2-methyl-1,1-dimethylethyl ester-2H-pyrrole-2-carboxylic acid-1-oxide (BMPO) to O_2^-, and 2,2,6,6-tetramethyl-1-piperidinyloxy (TEMPO) to $^1O_2$.

Enzymatic activities capacity of CMCO nanozymes partially sulfurized

The OPD as probe was used to assess the generation of ·OH in the presence of H_2O_2. CMCO (10 μg·mL^{-1}) reacted with NaHS·xH_2O (100 μg·mL^{-1}) for 10 min, and the products were collected by centrifugation and dispersed with the same volume deionized water. Products, H_2O_2 (1 mM) and OPD (1 mM) were added into PBS (pH = 6.5). After centrifugation at different time-point (0, 2, 4, 8, 12, 16, 20 min), the absorbance of the supernatant solution was recorded using UV-vis spectrophotometer.

The DPBF was used to assess the generation of $^1O_2$ and O_2^-. The products of CMCO (10 μg·mL^{-1}) reacting with NaHS (100 μg·mL^{-1}) for 10 min were mixed with DPBF (30 μg·mL^{-1}). After centrifugation at different time-point (0, 5, 10, 20, 30 min), the absorbance of the supernatant solution was recorded using UV-vis spectrophotometer.

The DTNB was used to assess the consumption of GSH. CMCO (10 μg·mL^{-1}) reacted with NaHS·xH_2O (100 μg·mL^{-1}) in PBS (pH = 6.5) for 10 min, and the products were collected by centrifugation and dispersed with the same volume deionized water. The products (0, 6.25, 12.5, 25, 37.5, 50, 100 μg·mL^{-1}) reacted with GSH (1 mM) for 10 min (complete reaction). After centrifugation, the supernatant were mixed with DTNB in PBS (pH = 6.5) for 10 min (complete reaction). The absorbance of the supernatant solution was recorded using UV-vis spectrophotometer.

Cu_2O, CMCO, and the products of CMCO reacting with NaHS for 10 min (50 μg·mL^{-1}) were added into PBS (pH = 6.5) in the presence H_2O_2 (4 mM). The real-time oxygen concentration was recorded using portable dissolved oxygen meter.

The MB was used to assess the generation of total ROS. CMCO (40 μg·mL^{-1}), H_2O_2 (1 mM), NaHS (100 μg·mL^{-1}) and MB (30 μg·mL^{-1}) were added into PBS (pH = 6.5). 1 W·cm^{-2} of 1064 nm irradiation, and reaction of CMCO with NaHS for 10 min. Different groups were divided according to the experiment design. After centrifugation at different time-point (5, 10, 20 min), the absorbance of the supernatant solution was recorded using UV-vis spectrophotometer.

Cell compatibility

1×10^4 CT26 and 8×10^3 L929 cells were seeded into 96 well plates for 24 h, respectively. The different concentrations (0, 2.5, 5, 10, 15, 20 μg·mL^{-1}) of CMCO / Cu_2O were added into medium for co-culture with cells for 24 h. The relative cell viabilities were detected by the standard 3-(4,5-dimethylthiazol-2-yl)-2,5-diphenyltetrazolium bromide (MTT) assay.

1×10^4 CT26 cells were seeded into 96 well plates for 24 h and then incubated with different concentrations (0, 2.5, 5, 10, 15, 20 μg·mL^{-1}) of CMCO. After co-culture for 4 h, each well was irradiated by 1064 nm laser with 1 W·cm^{-2} for 5 min. Cultured 24 h again, the relative cell viabilities were detected by MTT.

Live / dead cell staining analysis

5×10^4 CT26 cells were seeded into 24 well plates and grown for 24 h. Then the cells were treated with PBS, Cu_2O (20 μg·mL^{-1}) and CMCO (20 μg·mL^{-1}) for 4 h and further irradiated with or without 1064 nm laser (1 W·cm^{-2}, 5 min). After incubation for another 24 h, the cells were stained with calcein-AM and PI. The fluorescence was detected using an inverted

florescence microscope. The viable cells showed green fluorescence (λ_{ex} = 490 nm, λ_{em} = 515 nm), and the dead cells showed red fluorescence (λ_{ex} = 535 nm, λ_{em} = 617 nm).

Intracellular ROS detection

The intracellular ROS genaration was detected using ROS assay kit. 1×10^5 CT26 cells were seeded on coverslips in 24 well plates and grown for 24 h. And then, the cells were treated with PBS, Cu_2O (20 μg·mL^{-1}) and CMCO (20 μg·mL^{-1}) for 4 h. After removing the culture medium, the cells were incubated with DCFH-DA and Hoechest for 20 min at 37 °C. Washed using serum-free medium, the cells were further irradiated with or without 1064 nm laser (1 W·cm^{-2}, 5 min) and imaged by confocal microscopy.

Detection of intracellular GSH

The CT26 cells were seeded into culture dishs (D = 9 mm) and treated with PBS, NIR, CMCO and CMCO + NIR for 4 h. After incubation, the cells were counted and collected for 5×10^6. The relative GSH contents of different groups were detected using the reduced GSH assay kit.

Apoptosis and ferroptosis analysis

1×10^4 CT26 cells were seeded into 96 well plates for 24 h and then incubated with Ferrostain-1(10 μM) and Z-VAD-FMK (10 μM) for 2h. The cells were treated with CMCO (20 μg·mL^{-1}) for 4 h and further irradiated by 1064 nm laser (1 W·cm^{-2}, 5 min). After incubation for another 24 h, the relative cell viabilities were detected by MTT.

Cuproptosis analysis

8×10^3 CT26 cells were seeded into 96 well plates for 12 h and then incubated with FDX-1 siRNA (20 nM) for 24 h. The cells were treated with Ferrostain-1(10 μM) and Z-VAD-FMK (10 μM) for 2h. After that, the cells were treated with CMCO (20 μg·mL^{-1}) for 4 h and further irradiated by 1064 nm laser (1 W·cm^{-2}, 5 min). After incubation for another 24 h, the relative cell viabilities were detected by MTT

Detection of intracellular protein expression

The CT26 cells were seeded into 6 well plates for 24 h. To analyse GPX-4 expression, four experiment groups were designed as PBS, NIR, CMCO and CMCO + NIR. To analyse HSP70 expression, nine experiment groups were designed as PBS, NIR, 42℃, CMCO, CMCO + Ferrostatin-1 (Fer-1), CMCO + Z-VAD-FMK (zVAD), CMCO + NIR, CMCO + Fer-1 + NIR and CMCO + zVAD + NIR. To analyse FDX-1 expression, six experiment groups were designed as PBS, NIR, CMCO, siRNA, CMCO + siRNA and CMCO + siRNA + NIR. To analyse ferroptosis down-regulated HSP70 protein stress-induced by cuproptosis, six experiment groups were designed as PBS, NIR, siRNA, CMCO + siRNA, CMCO + siRNA +

NIR and CMCO + siRNA + NIR + Fer-1. To analyse oligmerization of DLAT protein, three experiment groups were designed as PBS, CMCO and CMCO + NIR. After the above treatment, the cells were washed by PBS and the proteins were collected by lyase buffer. The protein concentrations were quantified by BCA protein assay kit. And then, denatured proteins boiled in loading buffer were separated by SDS-PAGE, and transferred onto polyvinylidene fluoride (PVDF) membranes. The membranes were blocked with 5% skim milk for 1 h at room temperature, and immunoblotted with primary antibodies including Actin (1:10000, Proteintech), GPX-4 (1:2000, ImmunoWay), HSP70 (1:4000, ImmunoWay), FDX-1 (1:3000, Abcam) and DLAT (1:1000, ImmunoWay) at 4 °C overnight. After washed by TBST, the membranes were incubated with secondary antibodies (1:4000, ImmunoWay) for 1 h at room temperature. The protein bands were finally visualized with an enhanced chemiluminescence (ECL) substrate kit.

In vivo therapy effect

BALB/c mice bearing CT26 tumors were treated with different groups (PBS, NIR, Cu_2O, Cu_2O + NIR, CMCO, CMCO + NIR) via intratumor administration. PBS, Cu_2O or CMCO (200 μg, 100 mL) was injected intratumorally or intravenously on days 1, 4, 7. 1064 nm laser (1 $W \cdot cm^{-2}$) was used to irradiate the tumor site for 10 min (irradiation for 5 min, interruption for 5 min, and irradiation for other 5 min) after 10 min drugs injection, and the temperature was controlled within 42 °C. The tumor length (L), tumor width (W) and mice weight were measured every two days. The tumor volume (V) was calculated using the formula $V = L \times W^2 \times 0.52$. On day 15, the main organs (heart, liver, spleen, lung and kidney) and tumor tissues were removed after mice sacrifice under anesthesia. The organs were harvested and dissected to make paraffin section for further hematoxylin and eosin (H&E) staining. The excised tumors were harvested and dissected to make paraffin section for further H&E staining and terminal deoxynucleotidyl transferase dUTP nick labeling (TUNEL). In addition, the blood was collected from the eyeball for biochemistry assay.

Distribution and metabolism of CMCO in vivo

The healthy BALB/c mice were intravenously injected with CMCO (200 μg, 100 μL) and the products (200 μg, 100 μL) of CMCO sulfurized by NaHS for 1 h, respectively. Besides, the CT26 tumor-bearing mice were intravenously injected with CMCO (200 μg, 100 μL). The main organs were collected, weighed and dissolved in mixed solution of concentrated nitric acid and hydrogen peroxide (5:1). Mn and Cu elements of various samples were detected by ICP-MS.

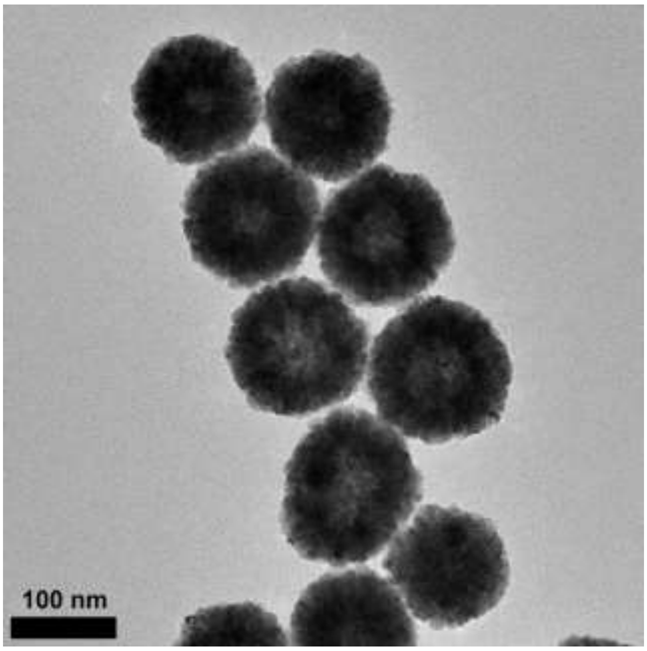

Figure S1. The TEM image of Cu_2O.

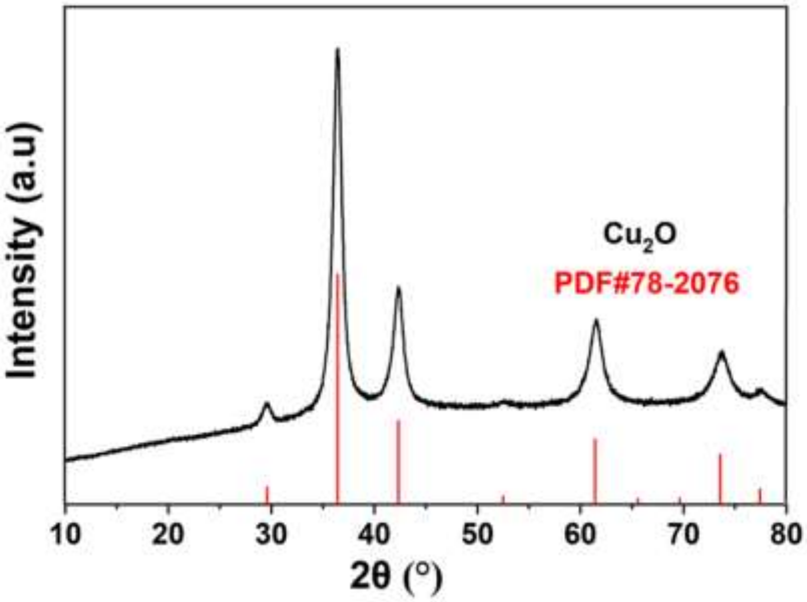

Figure S2. The XRD pattern of Cu_2O.

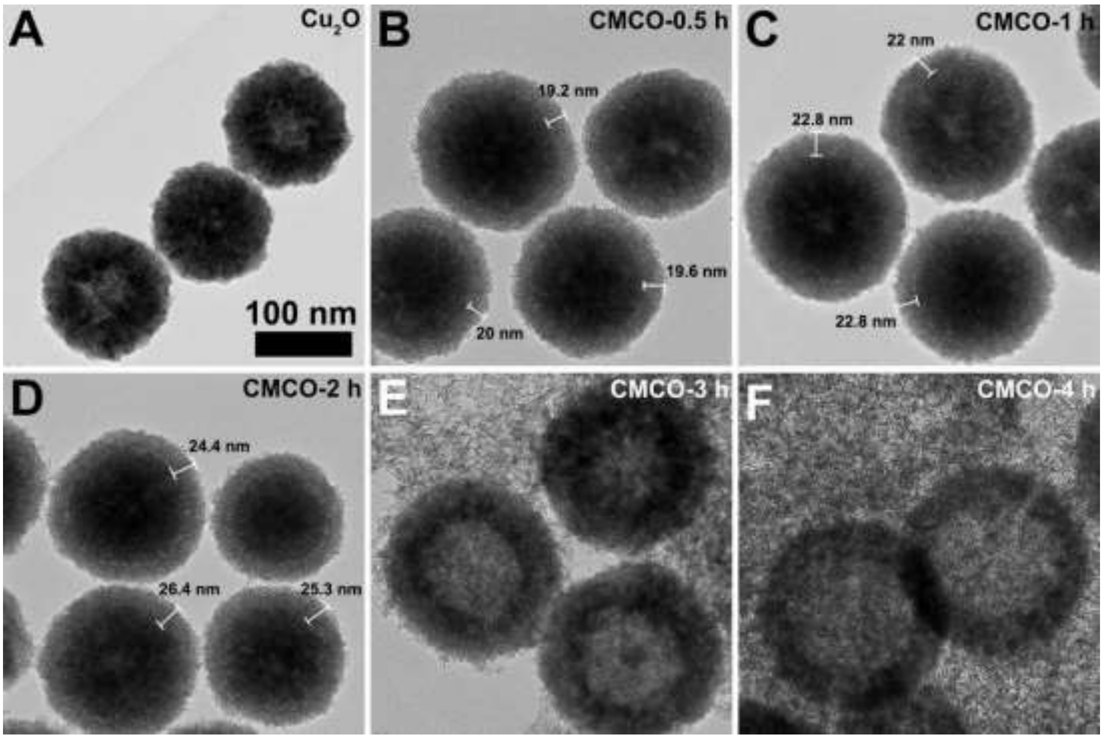

Figure S3. TME images of (A) Cu_2O and CMCO with different morphologies obtained by reaction of Cu_2O with $KMnO_4$ for (B) 0.5 h, (C) 1 h, (D) 2 h, (E) 3 h and (F) 4 h.

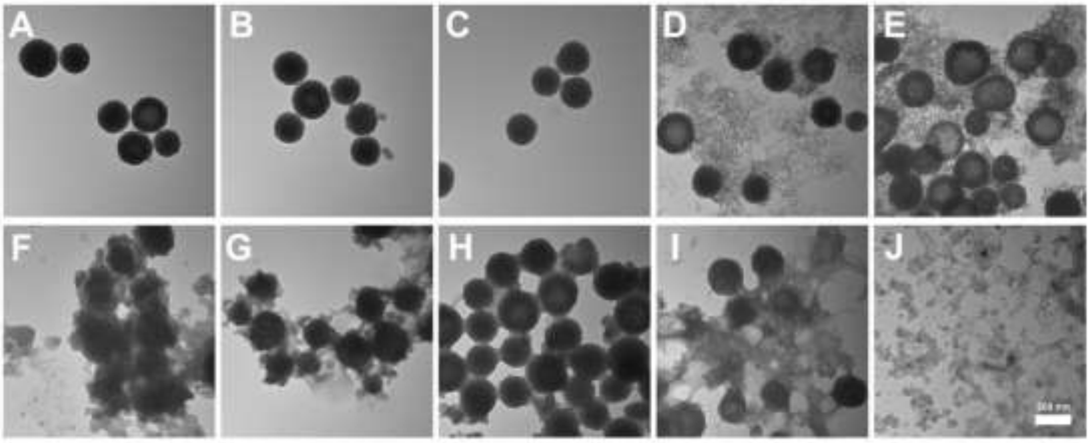

Figure S4. TME images of nanoparticles generated by the reaction of Cu_2O with $KMnO_4$ for (A) 0.5 h, (B) 1 h, (C) 2 h, (D) 3 h and (E) 4 h and (F-J) the corresponding products after being sulfurated by NaHS solution for 10 min.

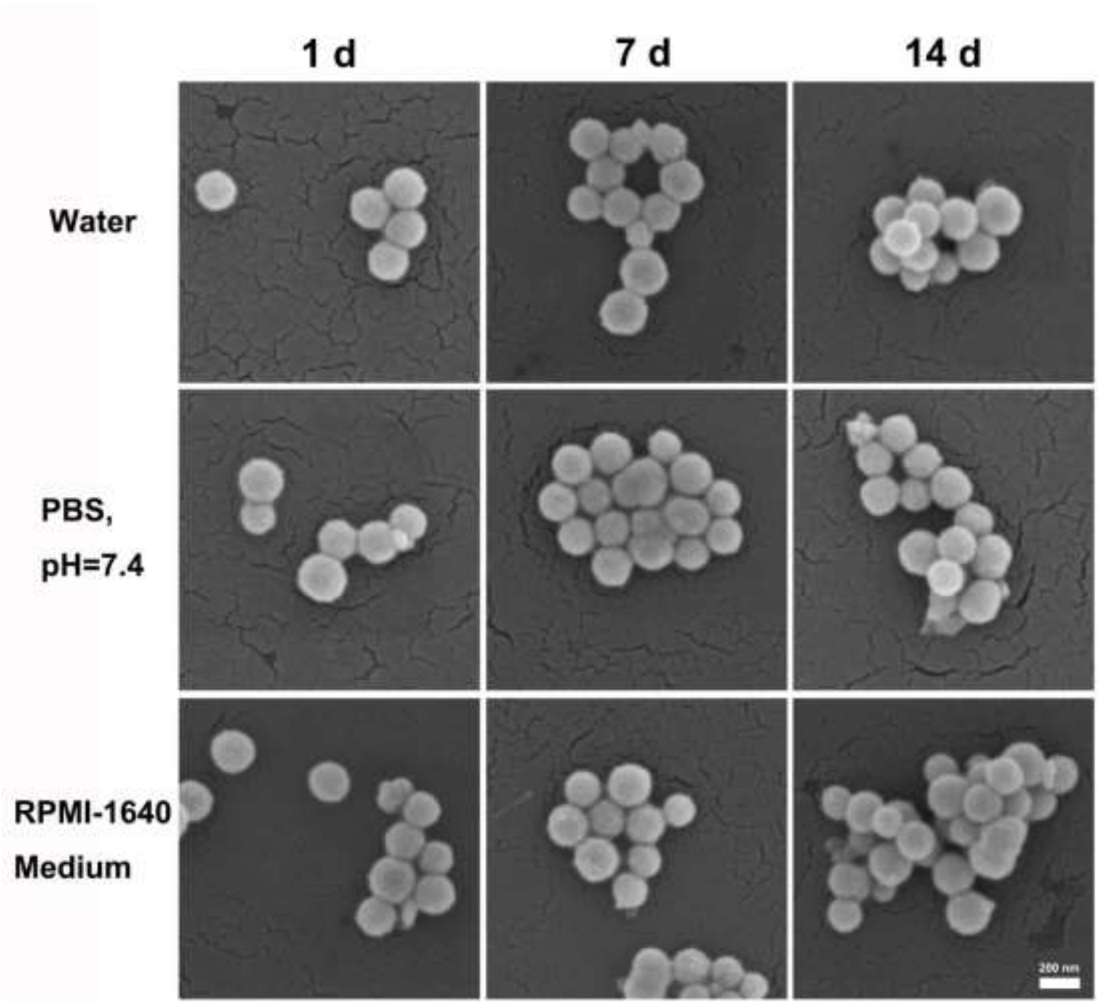

Figure S5. SEM images of CMCO nanozymes immersed in water, PBS (pH=7.4) and RPMI-1640 Medium for 1, 7 and 14 d.

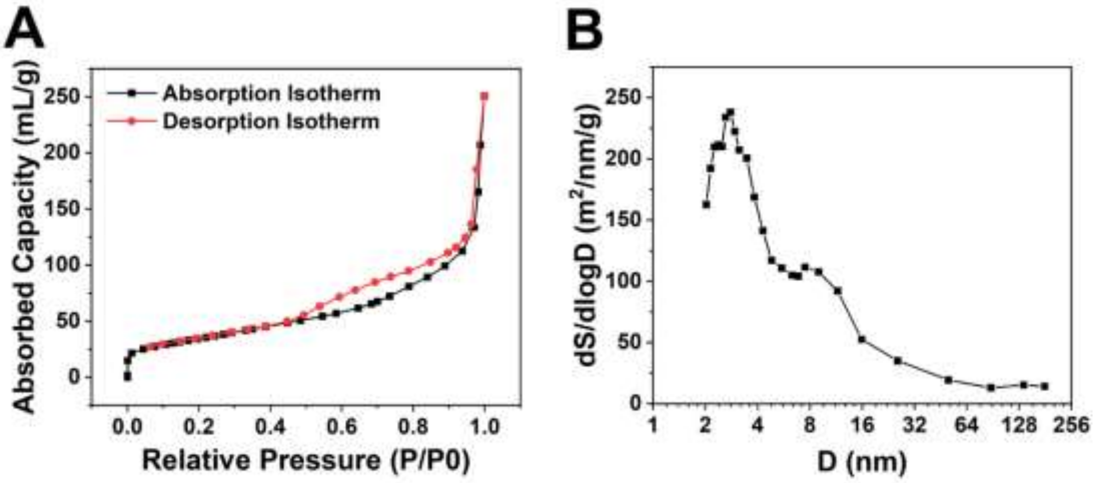

Figure S6. Nitrogen adsorption results for CMCO. (A) Absorption & desorbtion isotherm linear plot. (B)BJH (Absorption) pore area & pore size logarithm curve.

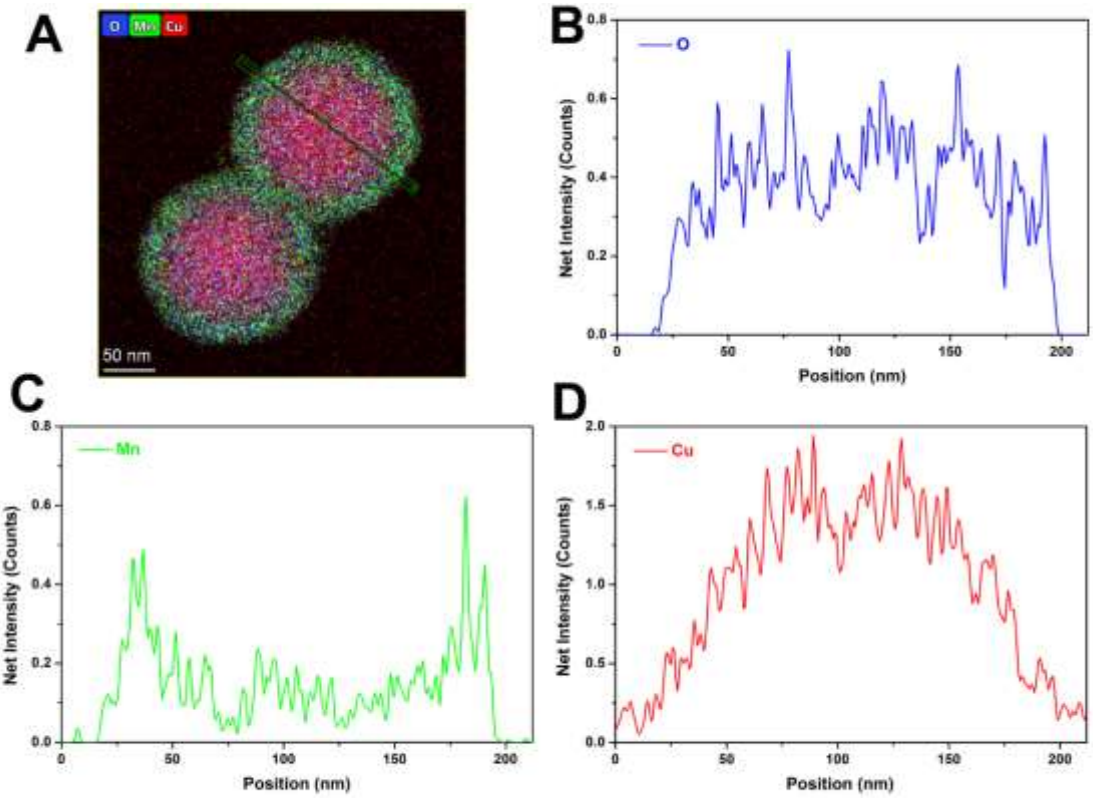

Figure S7. (A) Elemental mapping of CMCO. (B) O, (C) Mn and (D) Cu element content along with line scanning.

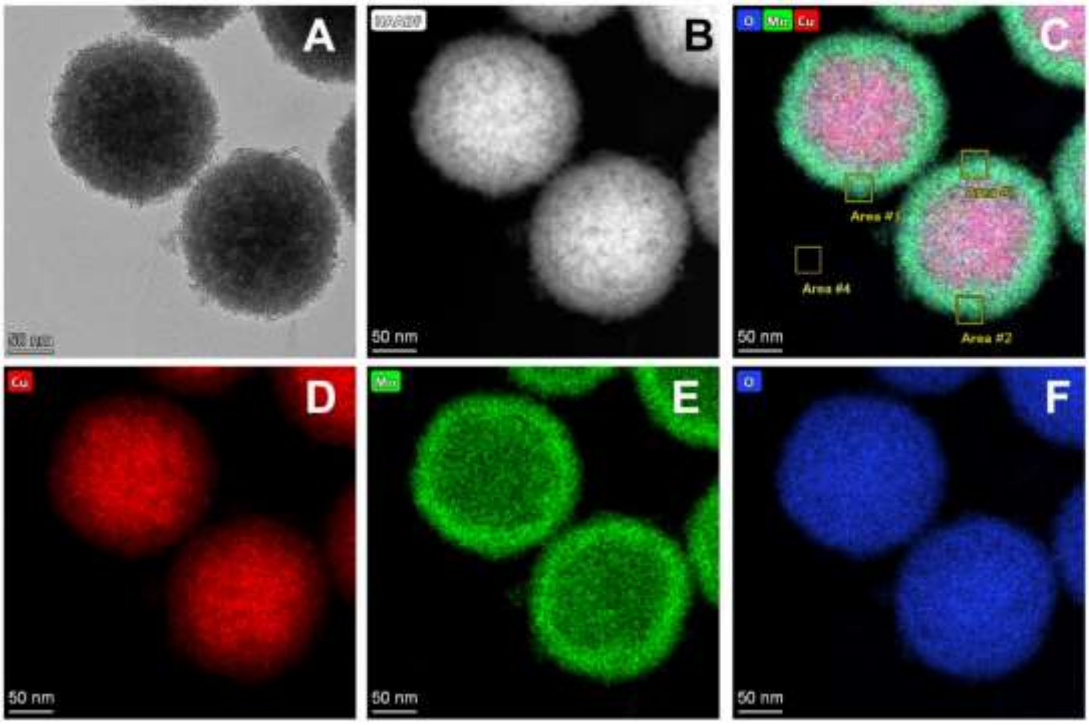

Figure S8. Elemental mapping of Cu, Mn and O of CMCO.

Table S1. The ratio of elements in the $Mn_3Cu_3O_8$ shell from the areas of Figure S6C.

Element	Area 1		Area 2		Area 3		Area 4	
	Ratio (%)	SD (%)	Ratio (%)	SD (%)	Ratio (%)	SD (%)	Ratio (%)	SD (%)
O	67.21	6.34	67.97	6.35	67.28	6.17	98.49	7.04
Mn	17.05	2.78	16	2.59	17	2.75	0.57	1.59
Cu	15.74	2.56	16.05	2.6	15.72	2.54	0.94	2.05

As shown in Table S1, the ratio of Cu and Mn was close to 1:1. However, due to the nickel oxide supporting film as the carrier stage, the O element in the blank could increase the proportion of O in the shell, so the true radio of O element can't be determined. Furthermore, the corresponding chemical formula and crystal form was analyzed based on the XRD pattern of the nanozyme (Figure 1D), assuming a Cu-Mn ratio of 1:1, and the shell layer was finally determined to be $Mn_3Cu_3O_8$.

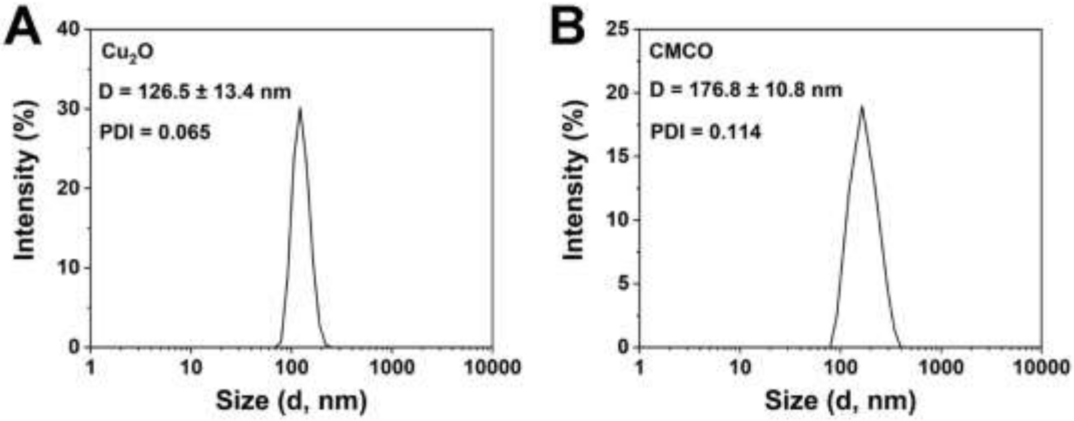

Figure S9. The size distribution of (A)Cu_2O and (B) CMCO.

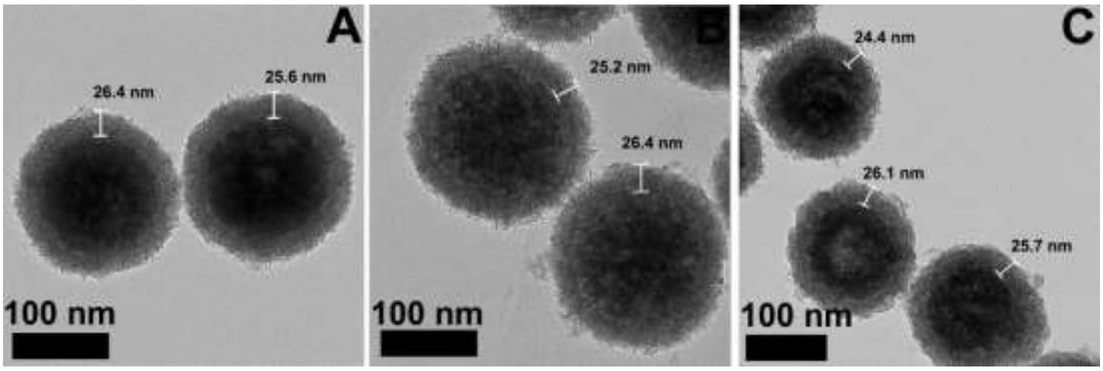

Figure S10. TME images of three batches of CMCO nanozymes for measuring the thickness of $Mn_3Cu_3O_8$ shell layer. It was calculated to be about 25.7 ± 0.7 nm.

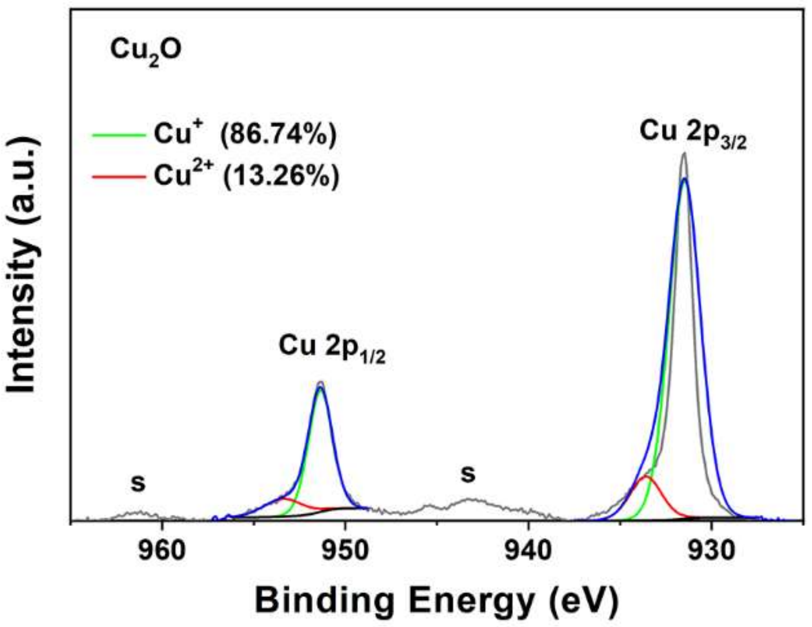

Figure S11. XPS high-resolution scans of Cu 2p in Cu_2O.

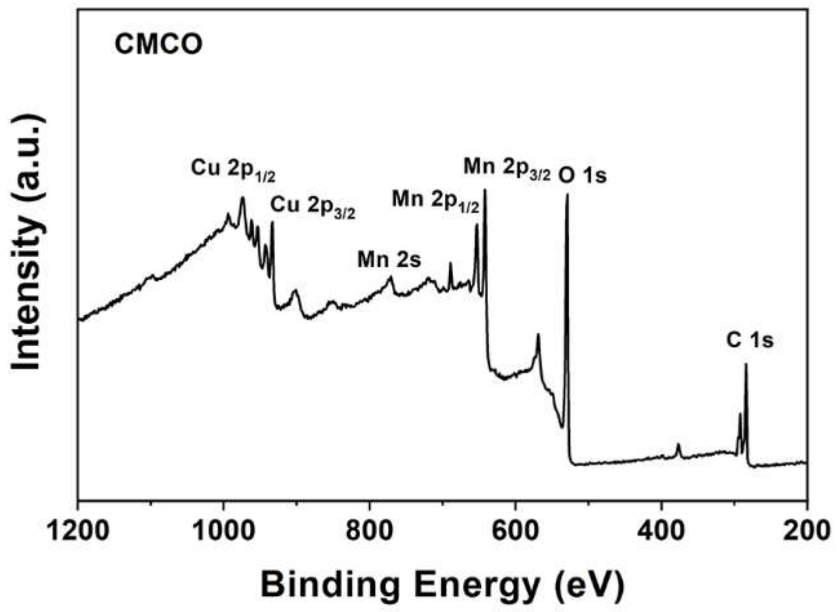

Figure S12. XPS spectra of CMCO.

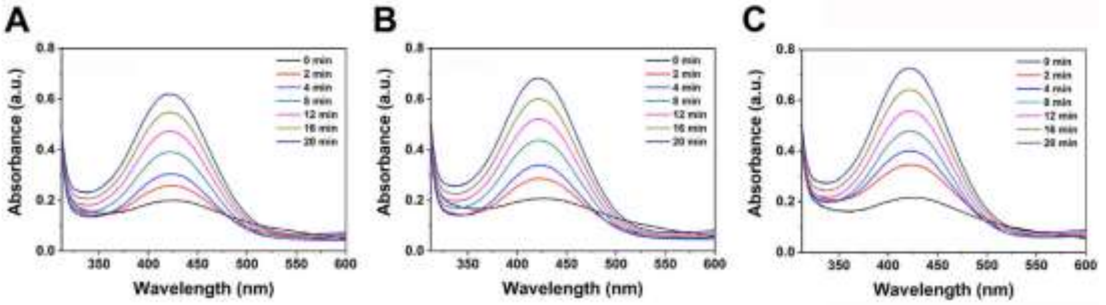

Figure S13. ·OH generation curves of CMCO (20 μg·mL^{-1}) obtained by reaction of Cu_2O with $KMnO_4$ for (A) 0.5 h, (B) 1 h and (C) 2 h with OPD (1 mM) as a probe in the presence of H_2O_2 (1 mM).

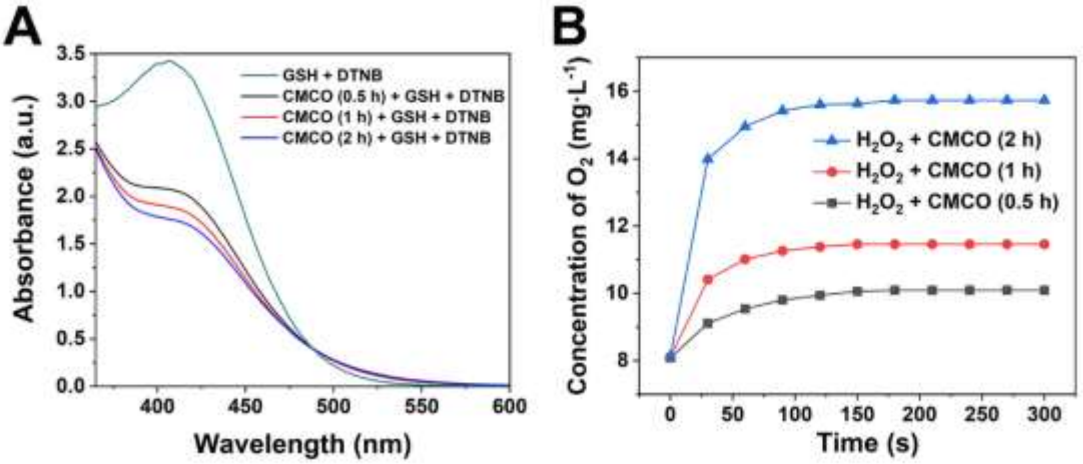

Figure S14. (A) GSH consumption curves of CMCO (30 μg·mL^{-1}) obtained by reaction of Cu_2O with $KMnO_4$ for 0.5 h, 1 h and 2 h with DTNB (100 μg·mL^{-1}) as a probe for 1 min. (B) O_2 generation cures of CMCO (30 μg·mL^{-1}) obtained by reaction of Cu_2O with $KMnO_4$ for 0.5 h, 1 h and 2 h in the presence of H_2O_2 (4 mM).

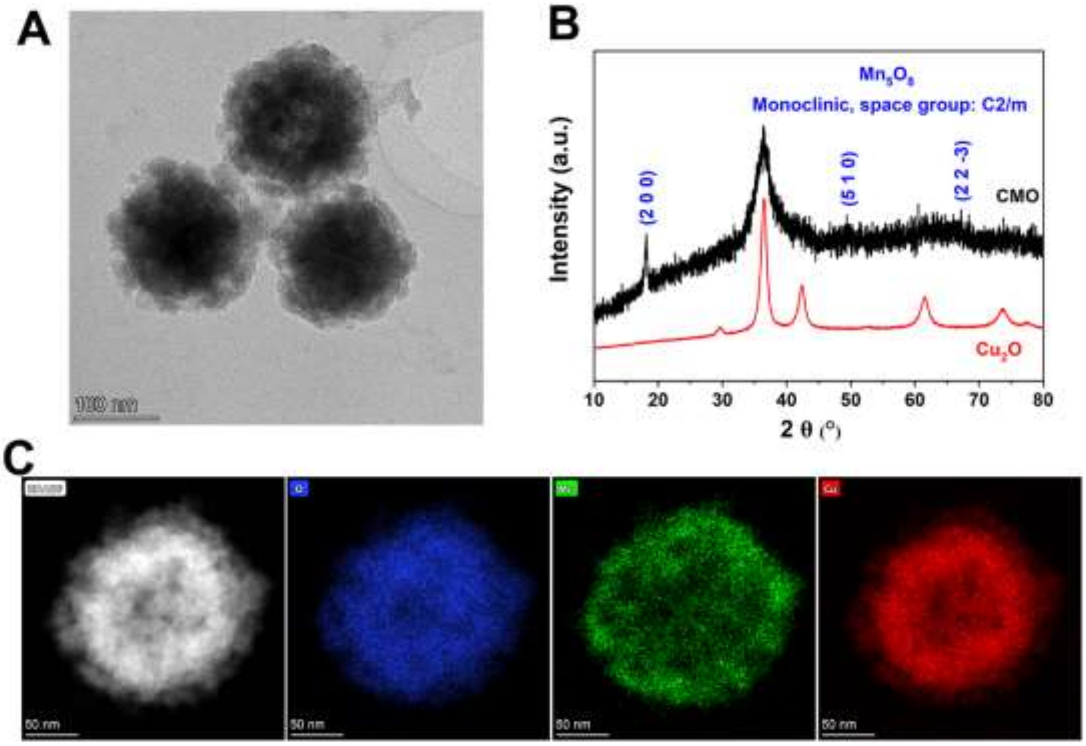

Figure S15. (A) TEM image, (B) XRD patten and (C) Elemental mapping of CMO.

The structure of CMO was similar to CMCO, which both were core-shell (Figure S15A). Differently, the shell of CMO was composed of monoclinic Mn_5O_8 (space group: C2/m) (Figure S15B). The elements of Cu, Mn and O were similarly distributed in different parts over the whole core-shell structure of CMO. O element homogeneously distributed on the core and shell, while Mn mainly distributed on the shell and Cu element mostly distributed on the core, respectively (Figure S15C).

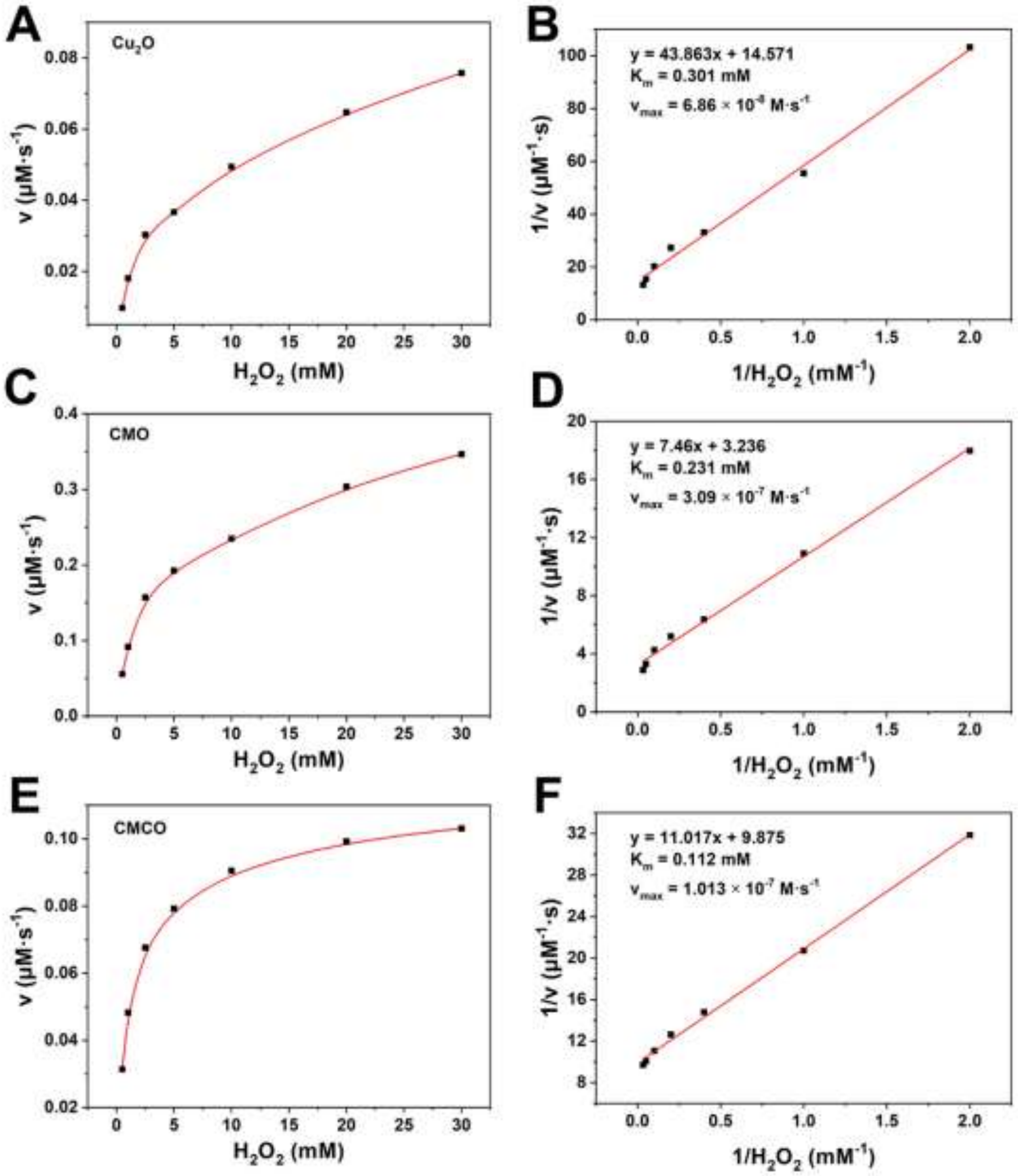

Figure S16. Michaelis-Menten kinetic analysis of POD-like activities for (A) Cu_2O, (C) CMO and (E) CMCO with H_2O_2 as a substrate. Lineweaver-Burk plot of POD-like activities for (B) Cu_2O, (D) CMO and (F) CMCO with H_2O_2 as a substrate.

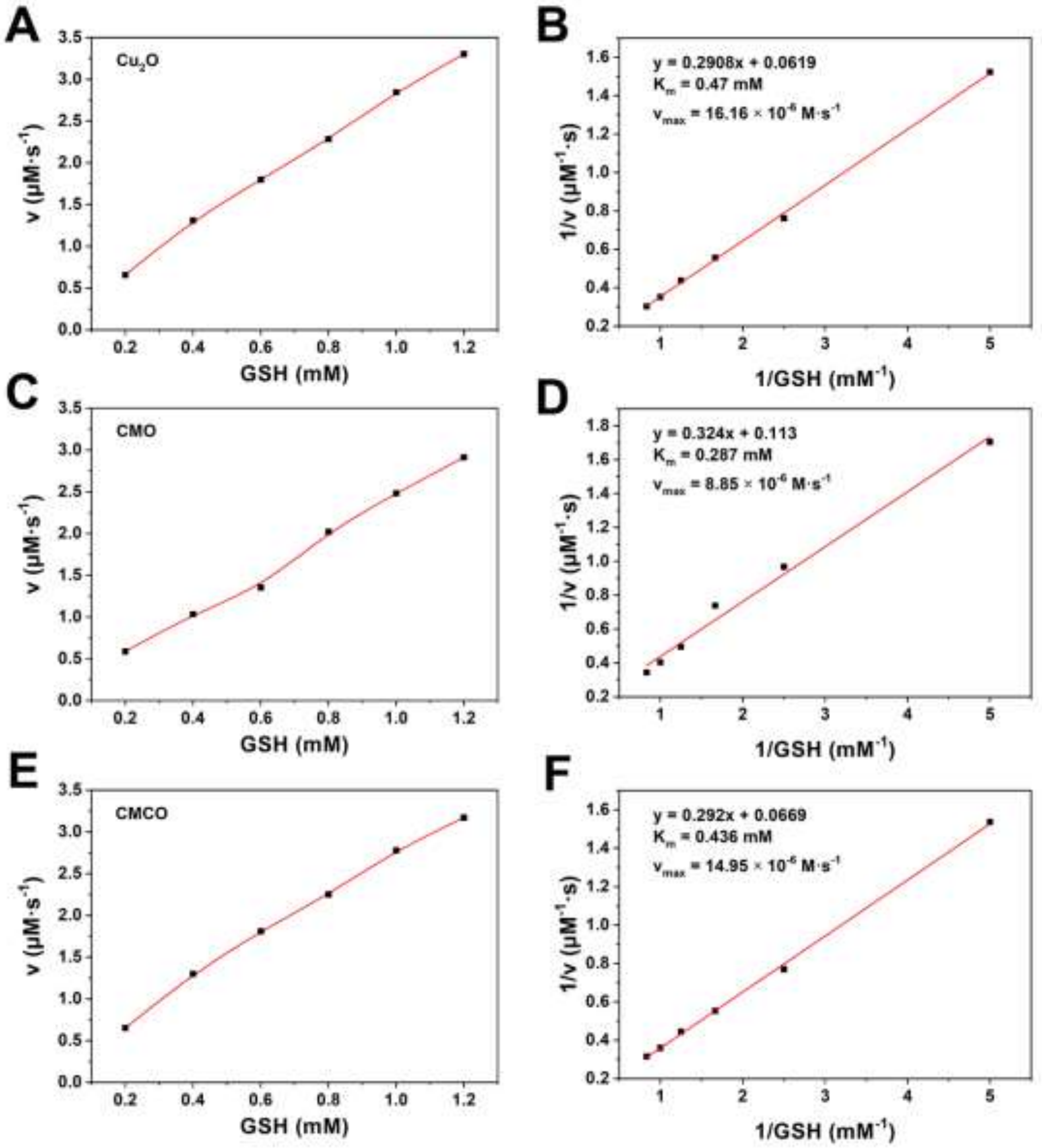

Figure S17. Michaelis-Menten kinetic analysis of GSHOx-like activities for (A) Cu_2O, (C) CMO and (E) CMCO with GSH as a substrate. Lineweaver-Burk plot of GSHOx-like activities for (B) Cu_2O, (D) CMO and (F) CMCO with GSH as a substrate.

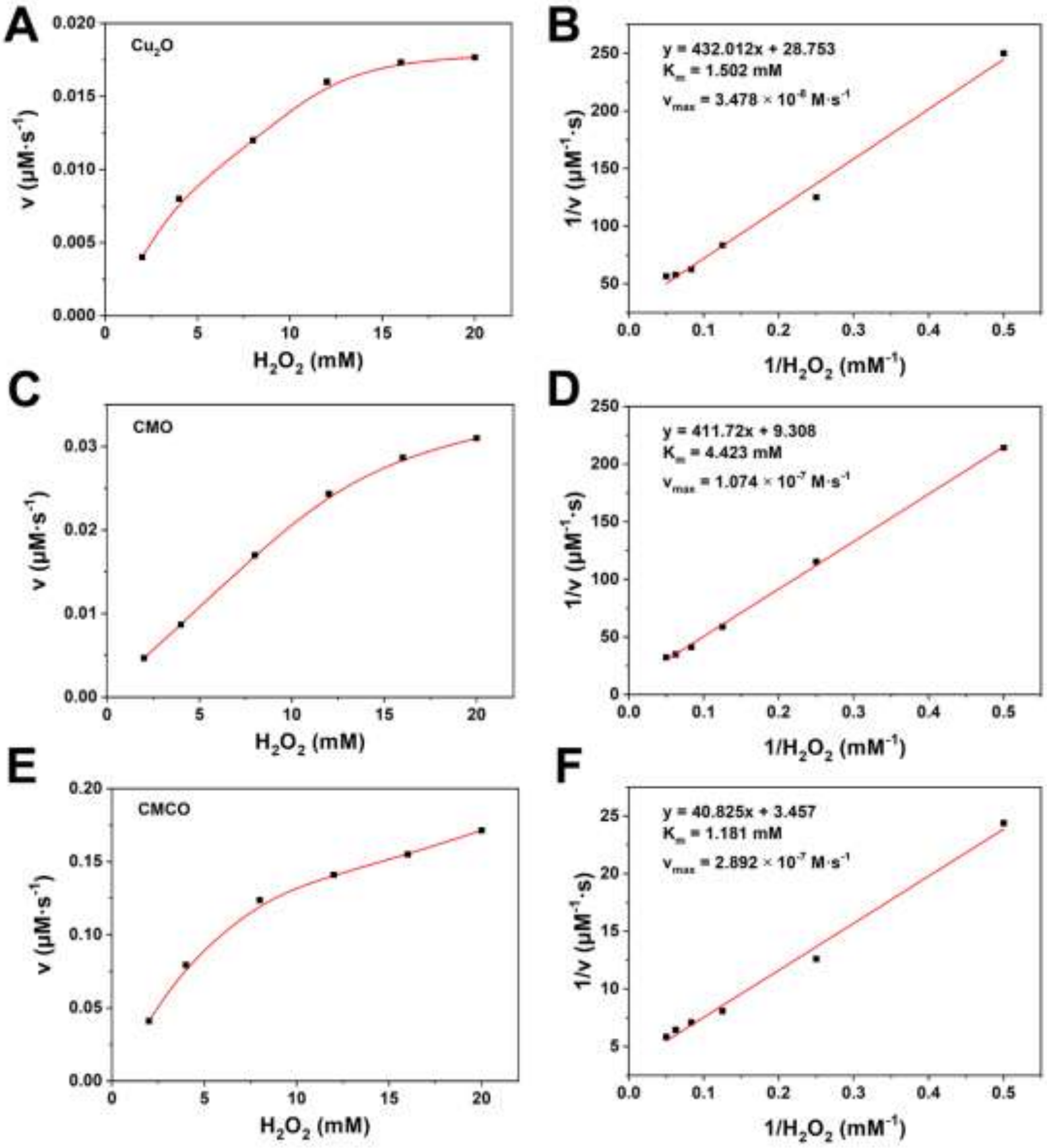

Figure S18. Michaelis-Menten kinetic analysis of CAT-like activities for (A) Cu_2O, (C) CMO and (E) CMCO with H_2O_2 as a substrate. Lineweaver-Burk plot of CAT-like activities for (B) Cu_2O, (D) CMO and (F) CMCO with H_2O_2 as a substrate.

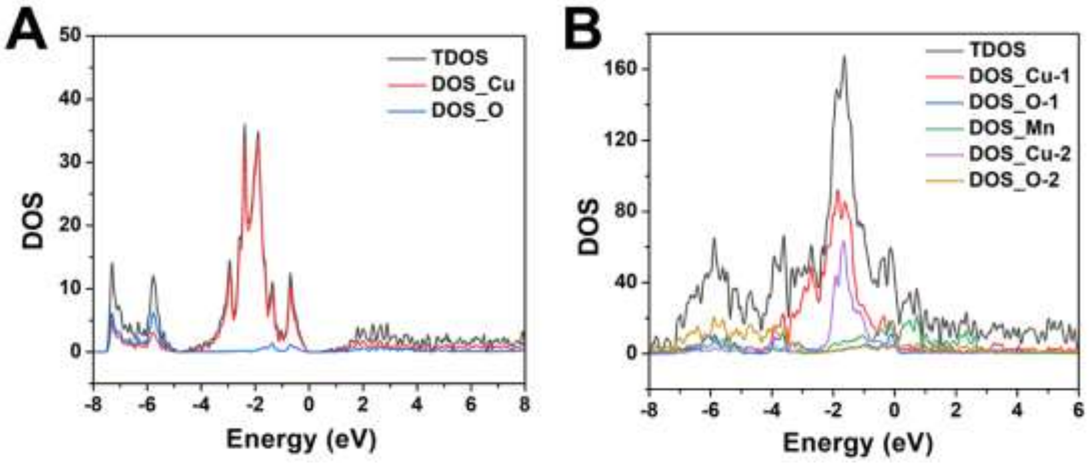

Figure S19. The densities of states of (A) Cu_2O and (B) CMCO nanozymes.

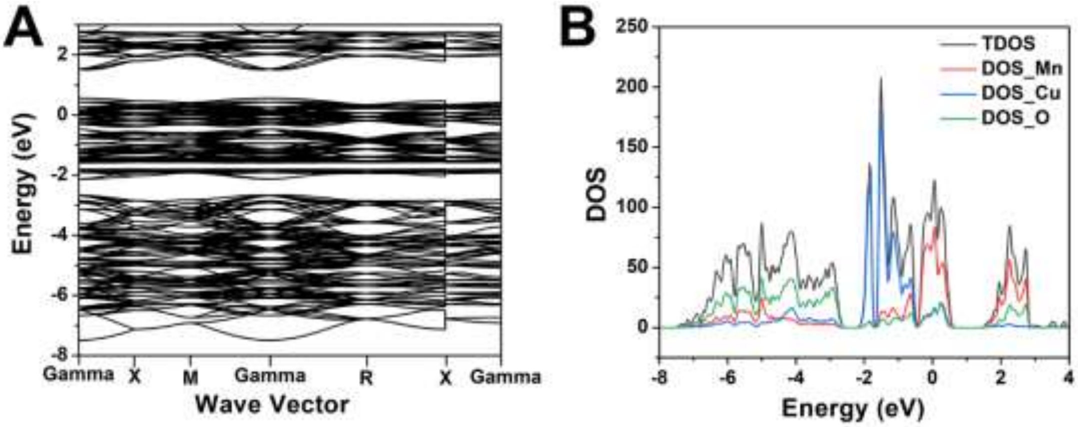

Figure S20. (A) Baseband calculation results and (B) densities of states of $Mn_3Cu_3O_8$ shell of nanozymes.

GSHOx-like activity:

$Cu^{2+}/Mn^{3+/4+} + GSH \rightarrow Cu^{+}/Mn^{2+} + GSSG$

CAT-like activity:

$Cu^{2+}/Mn^{3+/4+} + H_2O_2 \rightarrow Cu^{+}/Mn^{2+} + O_2$

POD-like activity:

$Cu^{+}/Mn^{2+} + H_2O_2 \rightarrow Cu^{2+}/Mn^{3+/4+} + \cdot OH + OH^-$

Figure S21. GSHOx-like, CAT-like, and POD-like activities catalyzing reactions of nanozymes.

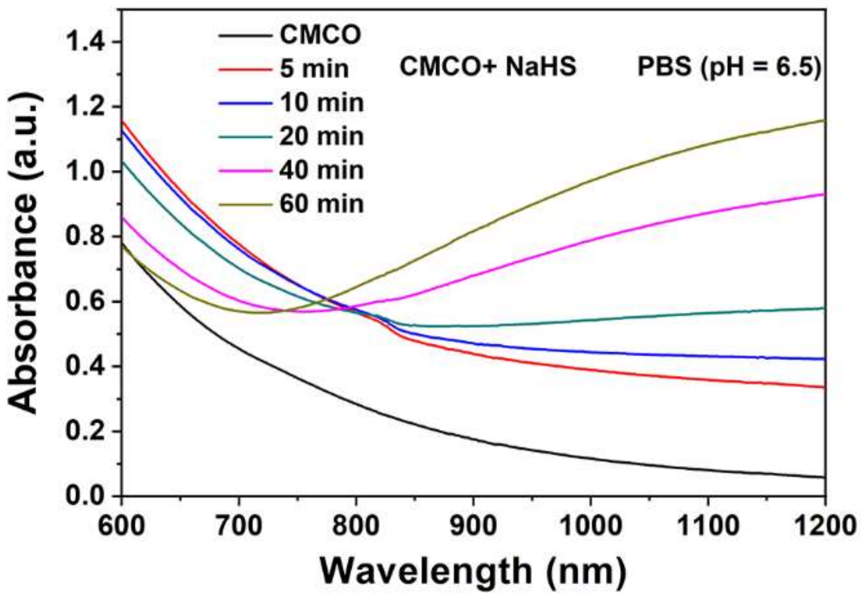

Figure S22. Vis-NIR absorption spectra of CMCO (100 μg·mL^{-1}) in the presence of NaHS (200 μg·mL^{-1}) in PBS (pH = 6.5) at different time.

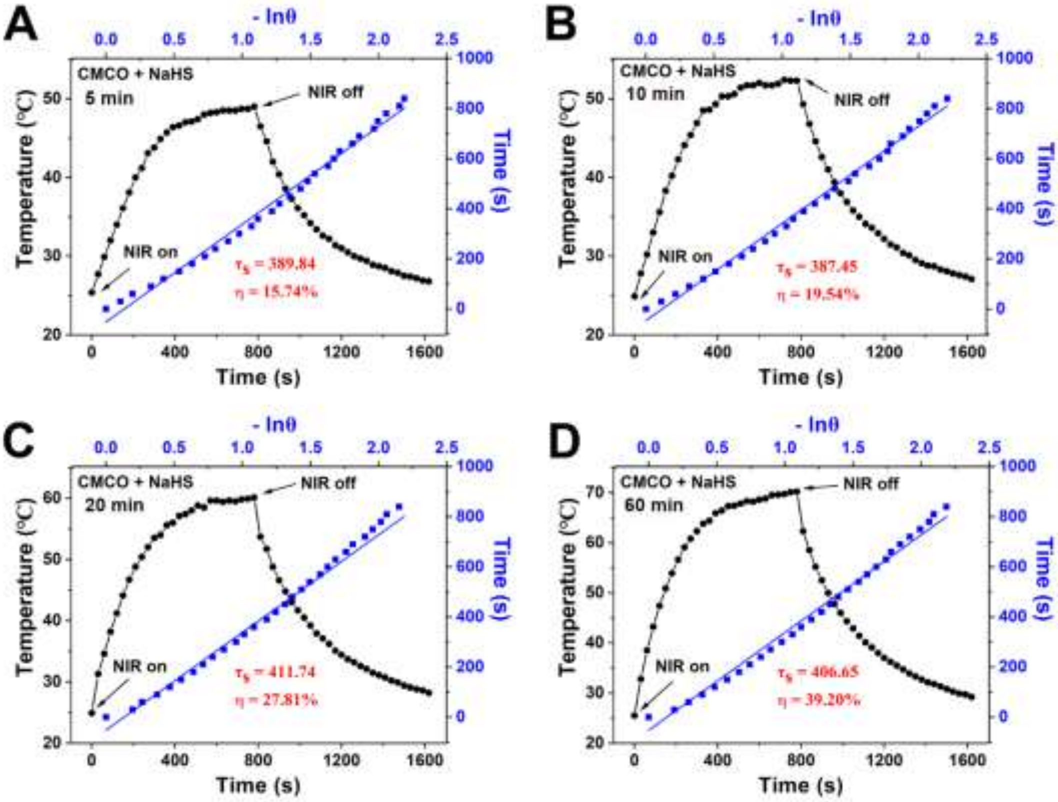

Figure S23. The photothermal conversion efficiency of CMCO (100 μg·mL^{-1}) under 1064 nm irradiation (1 W·cm^{-2}) after reaction with NaHS (200 μg·mL^{-1}) for (A) 5 min, (B) 10 min, (C) 20 min and (D) 60 min.

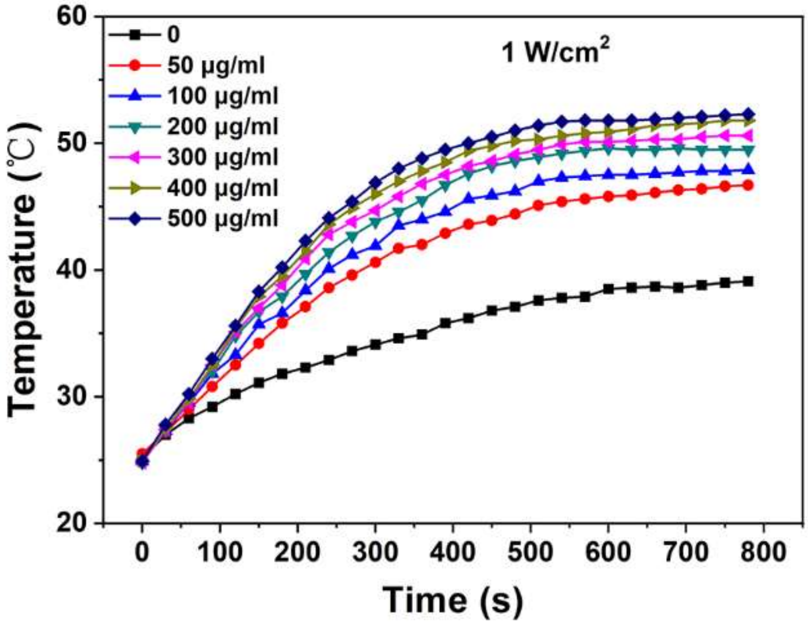

Figure S24. The heating curve of the products of CMCO (100 μg·mL^{-1}) reacting with different concentration NaHS for 10 min under 1064 nm irradiation (1 W·cm^{-2}).

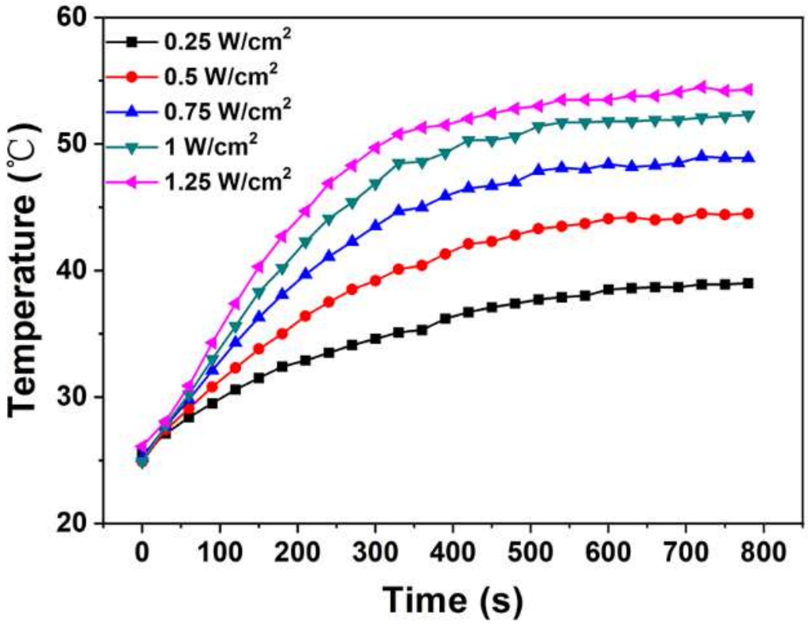

Figure S25. The heating curve of the products of CMCO (100 μg·mL^{-1}) reacting with NaHS (200 μg·mL^{-1}) for 10 min under 1064 nm irradiation with different power density.

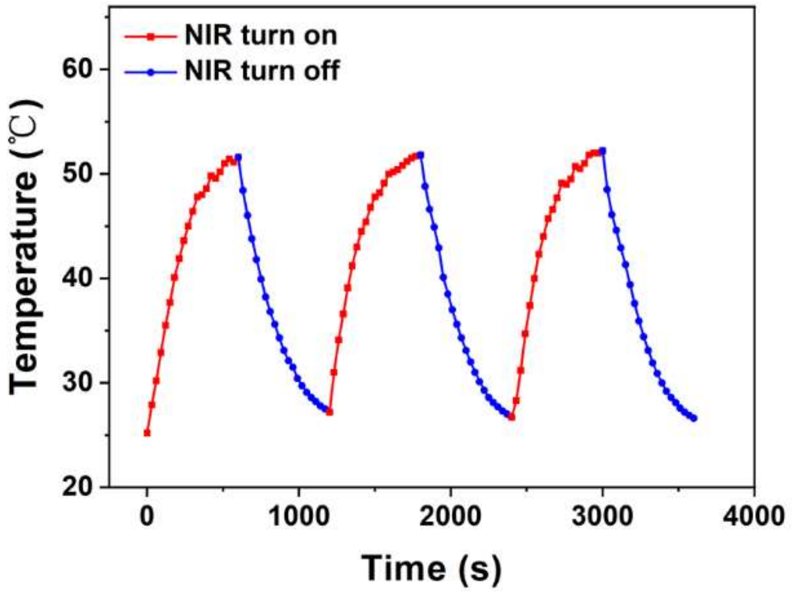

Figure S26. The photothermal conversion cycling test of the products of CMCO (100 μg·mL^{-1}) reacting with NaHS (200 μg·mL^{-1}) for 10 min under 1064 nm irradiation (1 W·cm^{-2}) (heating 10 min and cooling 10 min for one cycle).

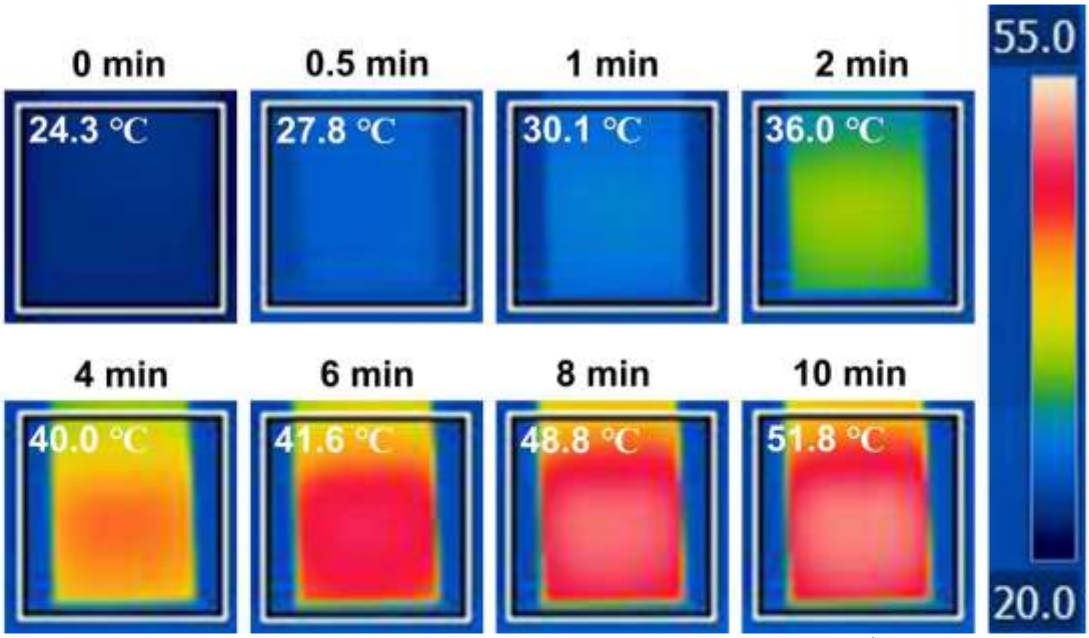

Figure S27. The thermal images of the products of CMCO (100 μg·mL^{-1}) reacting with NaHS (200 μg·mL^{-1}) for 10 min under 1064 nm irradiation (1 W·cm^{-2}) at different time.

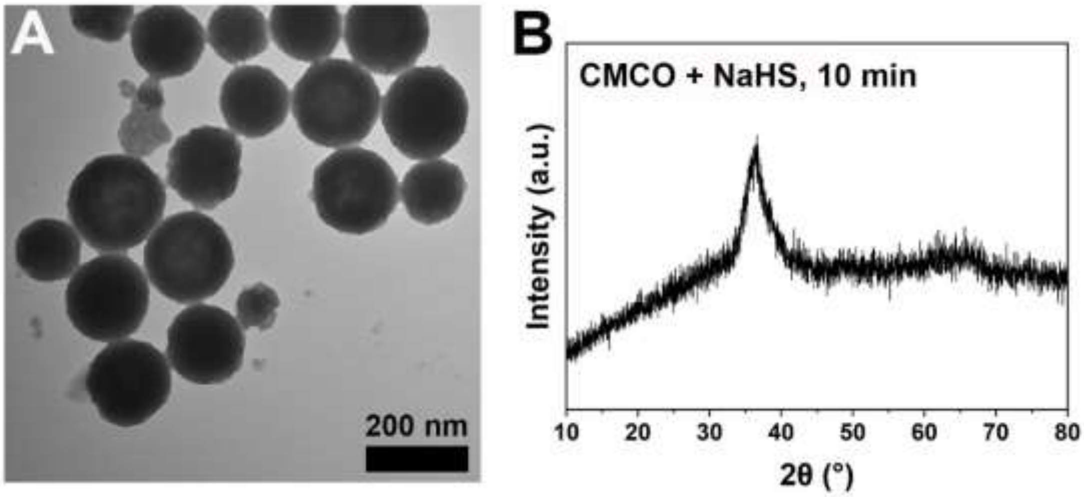

Figure S28. (A) TEM and (B) XRD of the products of CMCO (100 μg·mL^{-1}) reacting with NaHS (200 μg·mL^{-1}) for 10 min.

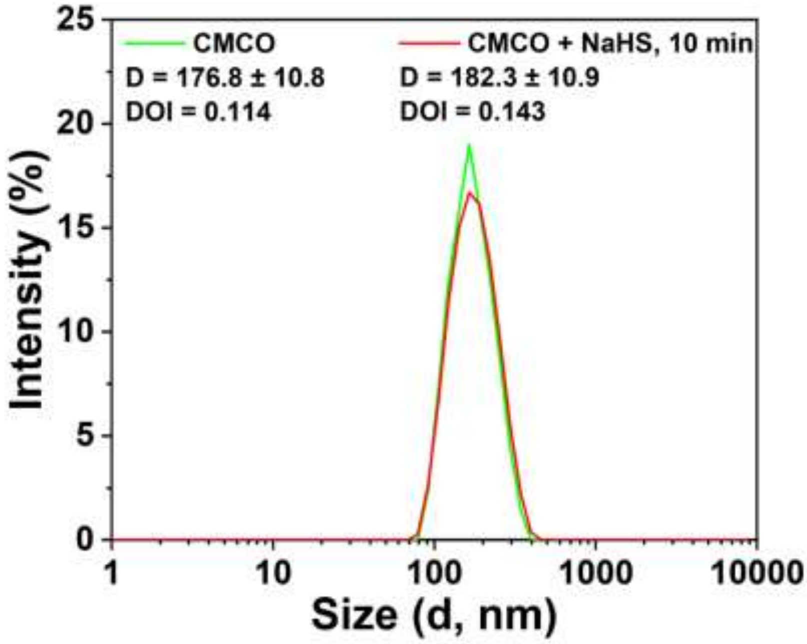

Figure S29. The size distribution of CMCO and the product of CMCO reacting with NaHS in PBS (pH = 6.5) for 10 min.

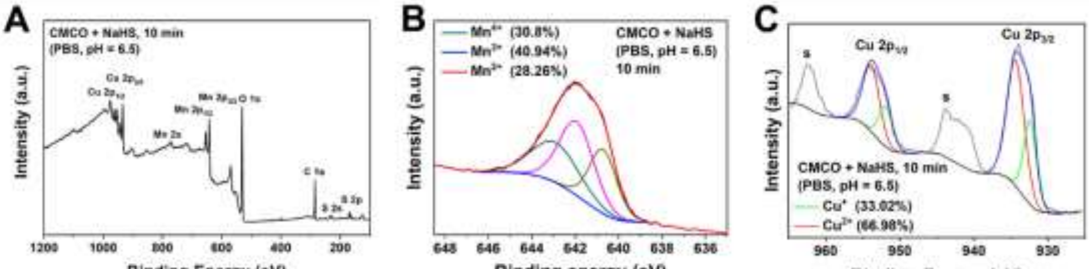

Figure S30. (A) XPS spectra of partially sulfurized CMCO. The XPS high-resolution scans of (B) Mn 2p and (C) Cu 2p in partially sulfurized CMCO.

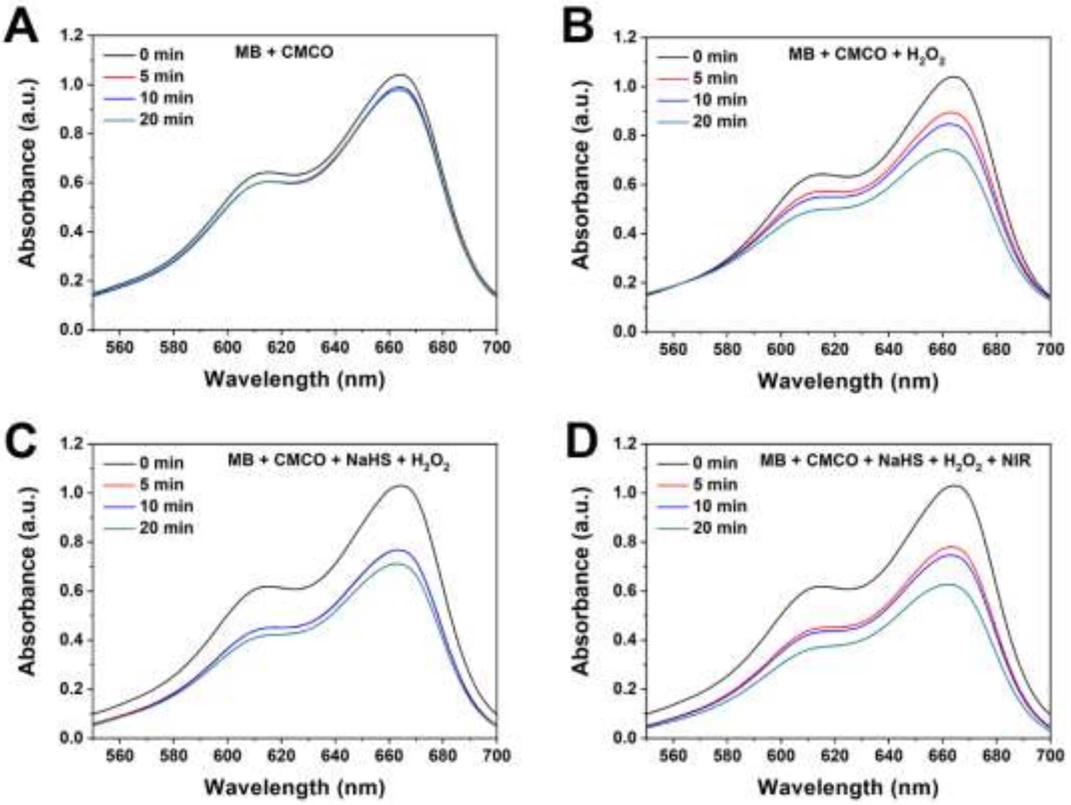

Figure S31. Degradation of MB due to ROS generation in the different groups, including (A) MB + CMCO, (B) MB + CMCO + H_2O_2, (C) MB + CMCO + NaHS + H_2O_2, (D) MB + CMCO + NaHS + H_2O_2 + NIR. Reaction of CMCO with NaHS in (C) and (D) for 10 min.

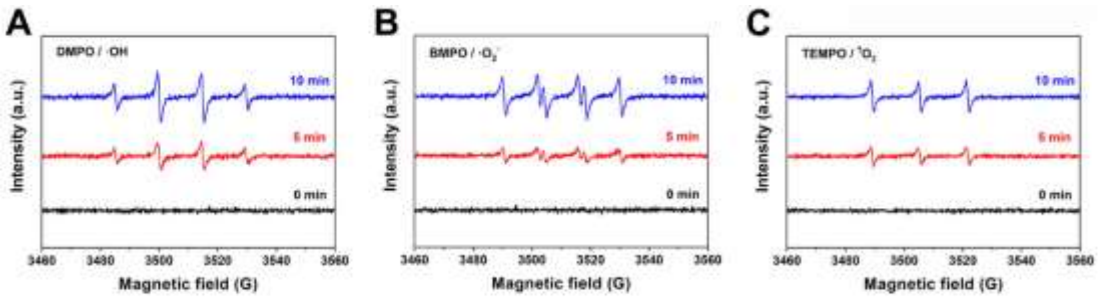

Figure S32. The ESR spectra of (A) DMPO/·OH, (B) TEMPO/$^1O_2$ and (C) BMPO/·$O_2^-$for the products of CMCO.

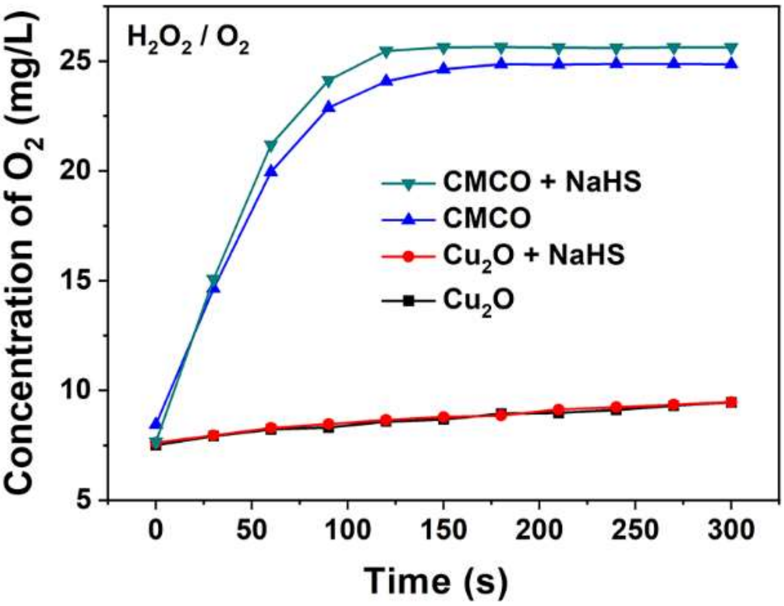

Figure S33. O_2 generation curve of Cu_2O, CMCO, and the products of CMCO reacting with NaHS for 10 min (50 μg·mL^{-1}) in the presence H_2O_2 (4 mM).

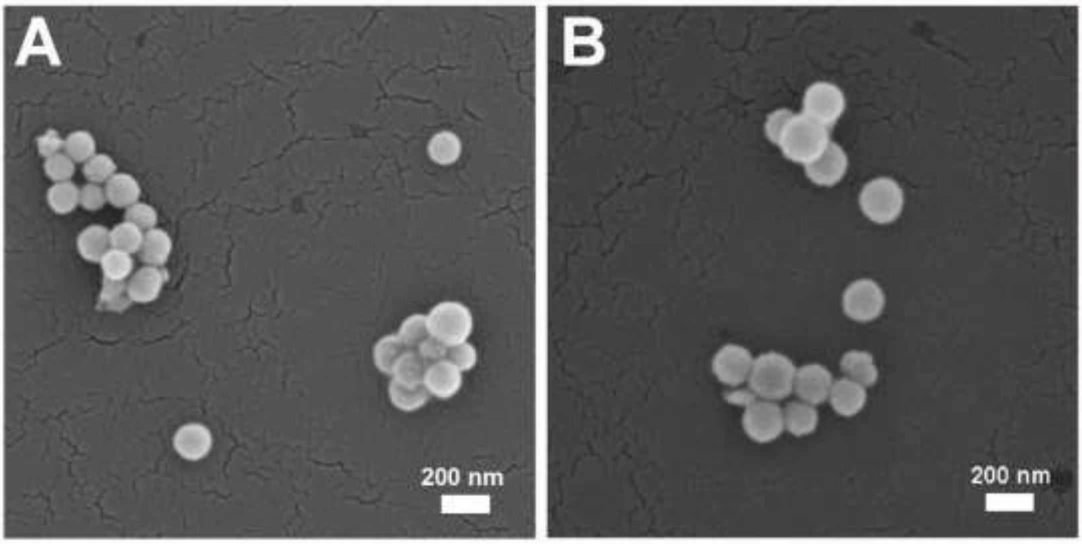

Figure S34. SEM images of (A) original CMCO (CMCO-BSA) and (B) BSA-coated CMCO (CMCO+BSA) after being immersed in PBS (pH = 7.4) for 15 d.

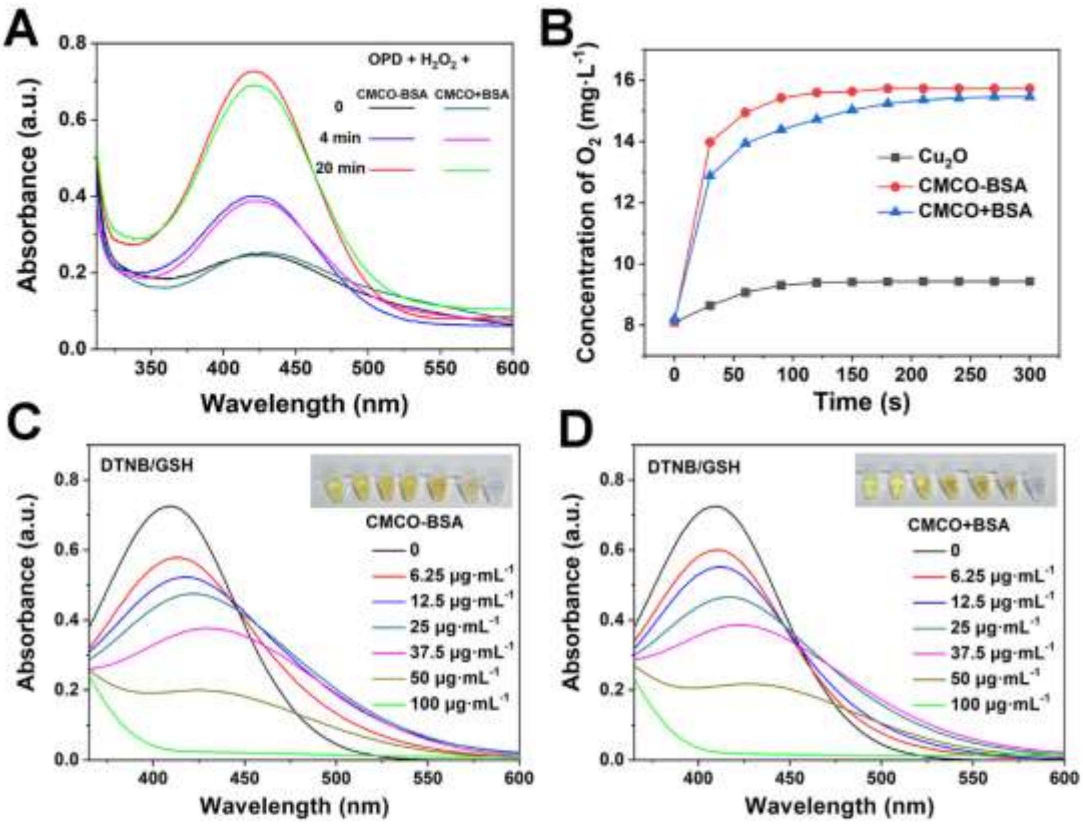

Figure S35. ·OH generation curves of CMCO-BSA and CMCO+BSA (20 μg·mL^{-1}) with OPD (1 mM) as a probe in the presence of H_2O_2 (1 mM) at different time. (B) O_2 generation cures of CMCO-BSA and CMCO+BSA (30 μg·mL^{-1}) in the presence of H_2O_2 (4 mM). GSH consumption curves with DTNB as a probe of (C) CMCO-BSA and (D) CMCO+BSA. Both nanozymes concentrations in illustration from left to right: 0, 6.25, 12.5, 25, 37.5, 50, 100 μg·mL^{-1}.

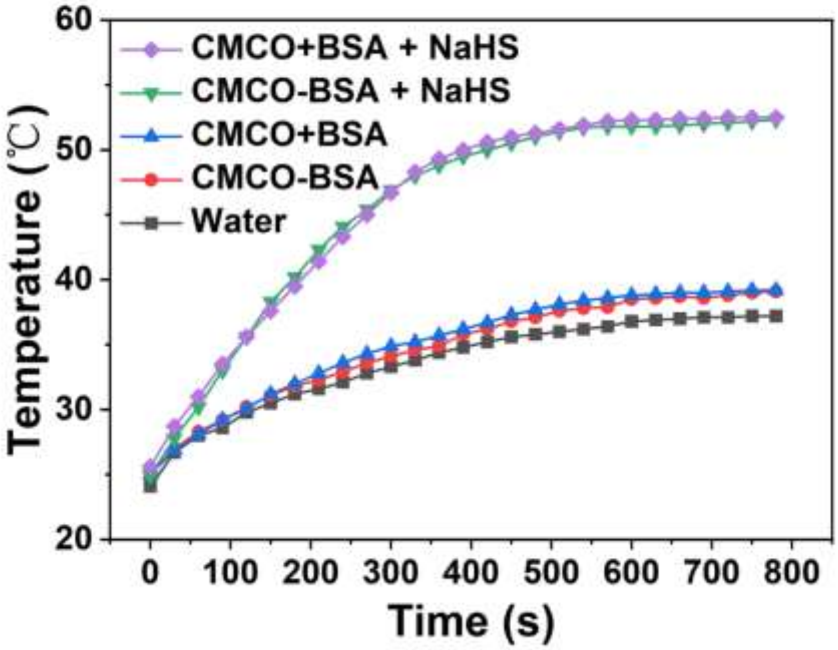

Figure S36. The heating curve of water, CMCO-BSA, CMCO+BSA, CMCO-BSA reacting with NaHS in PBS (pH = 6.5) for 10 min, CMCO+BSA reacting with NaHS in PBS (pH = 6.5) for 10 min.

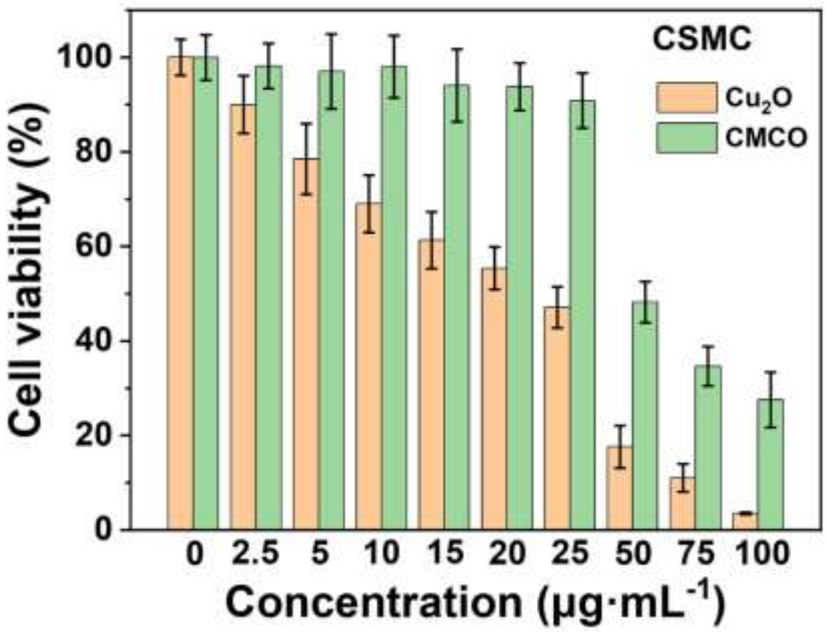

Figure S37. Cytotoxicity assessment on CSMC treated with different concentration of Cu_2O and CMCO nanozymes. Dates are presented as mean ± SD (n = 6).

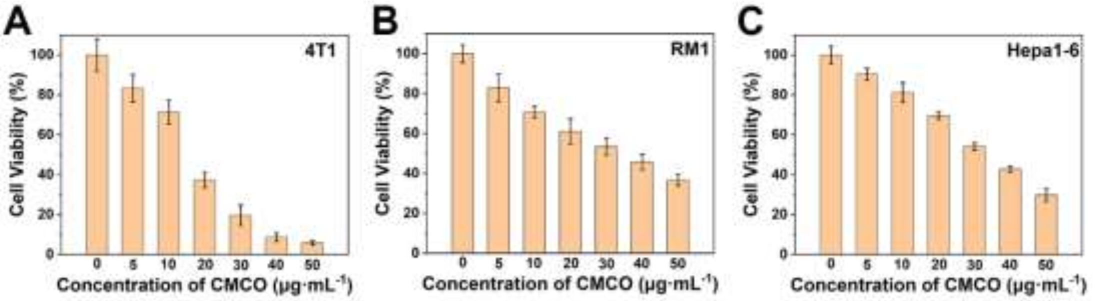

Figure S38. Cytotoxicity assessment on (A) 4T1 (mouse breast cancer cells), (B) RM1 (mouse prostate cancer cells) and (C) Hepa1-6 (mouse hepatocarcinoma cells) treated with different concentration of CMCO nanozymes. Dates are presented as mean ± SD (n = 6).

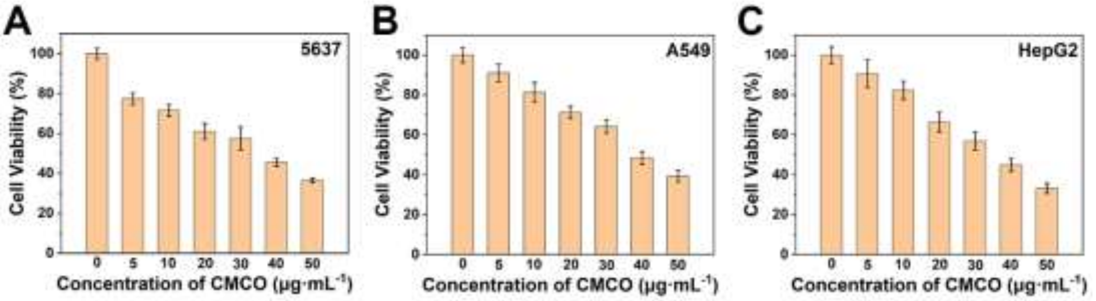

Figure S39. Cytotoxicity assessment on (A) 5637 (human bladder cancer cells), (B) A549 (human lung cancer cells) and (C) HepG2 (human hepatoma cells) treated with different concentration of CMCO nanozymes. Dates are presented as mean ± SD (n = 6).

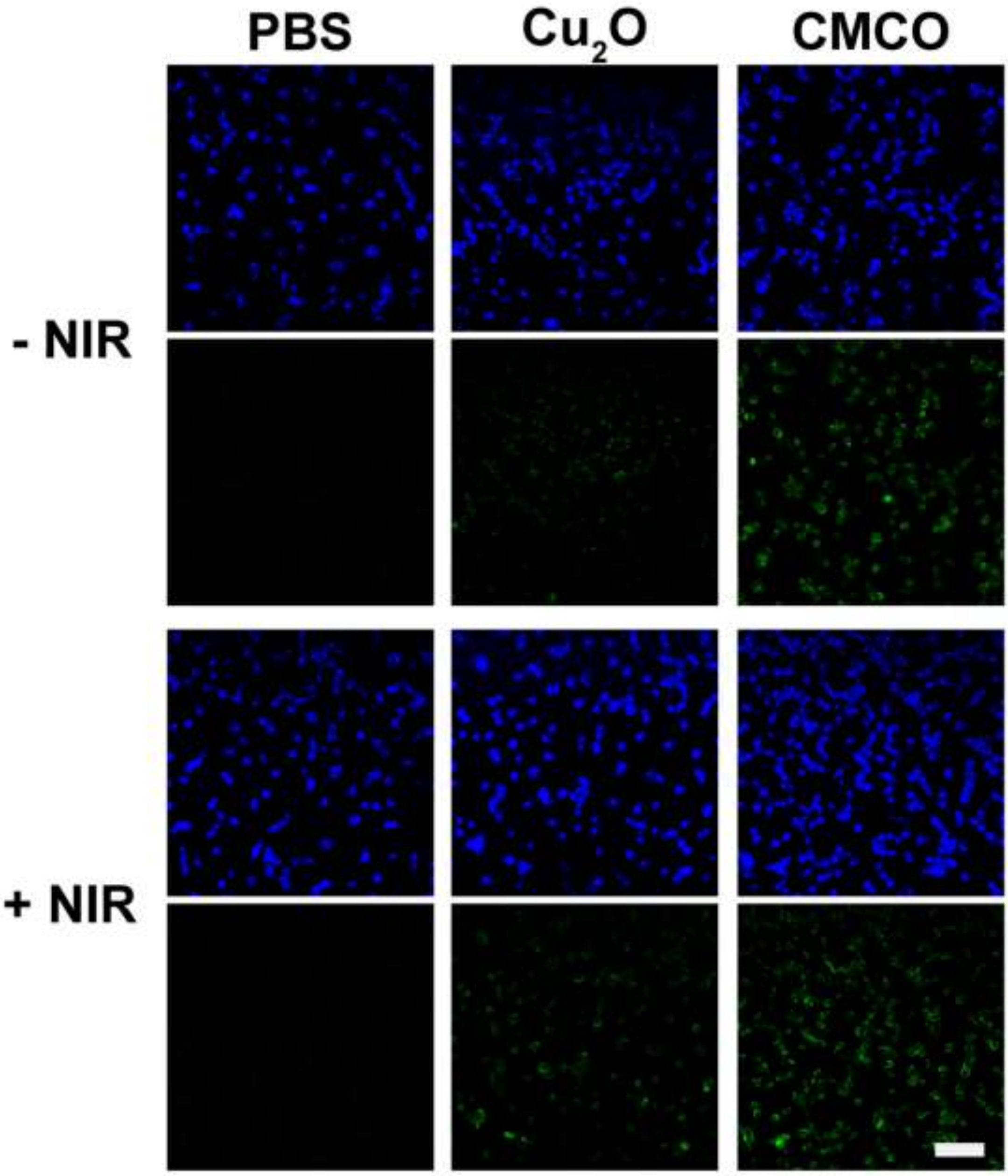

Figure S40. The detection of intracellular LPO after different treatments on CT26 cells (scale bar: 50 μm).

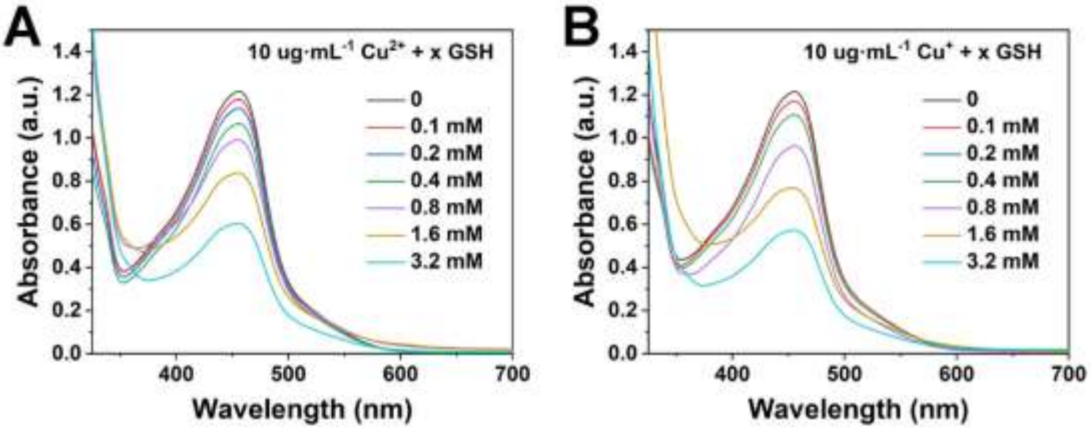

Figure S41. (A) Cu^{2+} and (B) Cu^{+} chelation curves with different concentration of GSH by using neocuproine spectrophotometry.

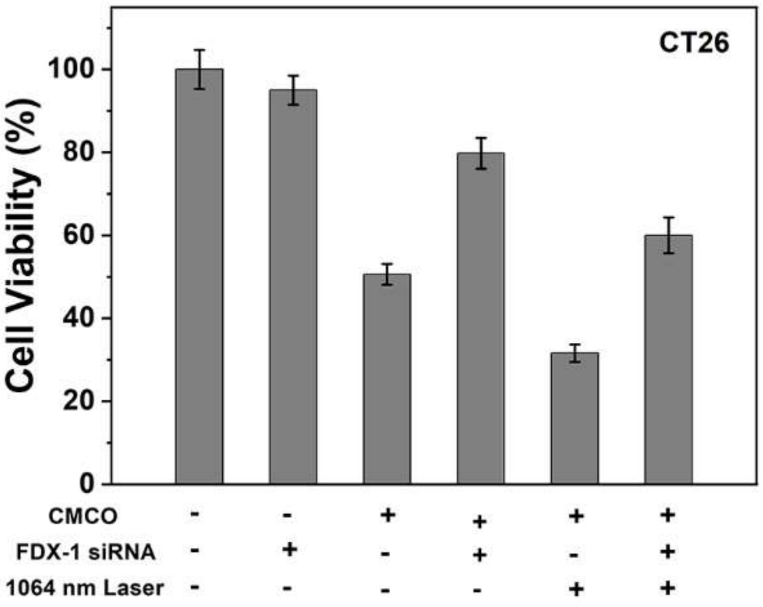

Figure S42. Cytotoxicity assessment on CT26 with different treatment. Dates are presented as mean ± SD (n = 6).

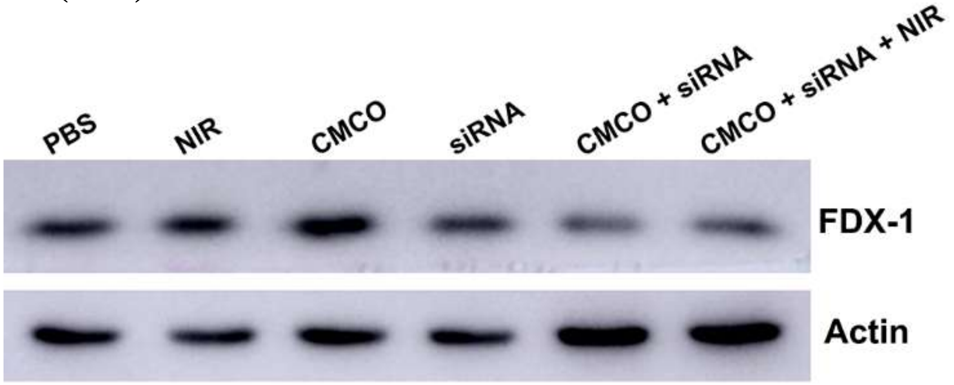

Figure S43. Western blot of FDX-1 after different treatments.

Table S2. Cytotoxicity assessment on CT26 cells with different treatments.

CMCO	-	-	-	+	+	+	+	+	+
Z-VAD-FMK	-	+	-	-	+	-	-	+	-
Ferrostain-1	-	-	+	-	-	+	-	-	+
NIR	-	-	-	-	-	-	+	+	+
Mean (%)	100	92.9	93	51.9	60.5	65.8	29.1	39.4	49.1
S.D. (%)	6.1	3.2	4.4	4.3	4.6	4.9	3.4	3.1	5.4

The death of CT26 cells was roughly induced by apoptosis, ferroptosis and cuproptosis. Z-VAD-FMK inhibits apoptosis, while Ferrostain-1 inhibits ferroptosis and apoptosis.

In the absence of NIR irradiation:

The cell viability was 51.9% in the group of CMCO, which indicates the effect of cuproptosis + ferroptosis + apoptosis for about 48.1% (100% - 51.9% = 48.1%). Record as:

$$\text{cuproptosis + ferroptosis + apoptosis} = 48.1\% \qquad \text{equation 7}$$

The cell viability was 60.5% in the group of CMCO + Z-VAD-FMK, which indicates the effect of cuproptosis + ferroptosis for about 39.5% (100% - 60.5% = 39.5%). Record as:

cuproptosis + ferroptosis = 39.5% equation 8

The cell viability was 65.8% in the group of CMCO + Ferrostain-1, which indicates the effect of cuproptosis for about 34.2% (100% - 65.8% = 34.2%). Record as:

cuproptosis = 34.2% equation 9

Therefore,

apoptosis = equation 7 - equation 8 = 48.1% - 39.5% = 8.6%

ferroptosis = equation 8 - equation 9 = 39.5% - 34.2% = 5.3%

cuproptosis = 34.2%

In the presence of NIR irradiation:

The cell viability was 29.1% in the group of CMCO + NIR, which indicates the effect of cuproptosis + ferroptosis + apoptosis for about 70.9% (100% - 29.1% = 70.9%). Record as:

cuproptosis + ferroptosis + apoptosis = 70.9% equation 10

The cell viability was 39.4% in the group of CMCO + Z-VAD-FMK + NIR, which indicates the effect of cuproptosis + ferroptosis for about 60.6% (100% - 39.4% = 60.6%). Record as:

cuproptosis + ferroptosis = 60.6% equation 11

The cell viability was 49.1% in the group of CMCO + Ferrostain-1 + NIR, which indicates the effect of cuproptosis for about 50.9% (100% - 49.1% = 50.9%). Record as:

cuproptosis = 50.9% equation 12

Therefore,

apoptosis = equation 10 - equation 11 = 70.9% - 60.6% = 10.3%

ferroptosis = equation 11 - equation 12 = 60.6% - 50.9% = 9.7%

cuproptosis = 50.9%

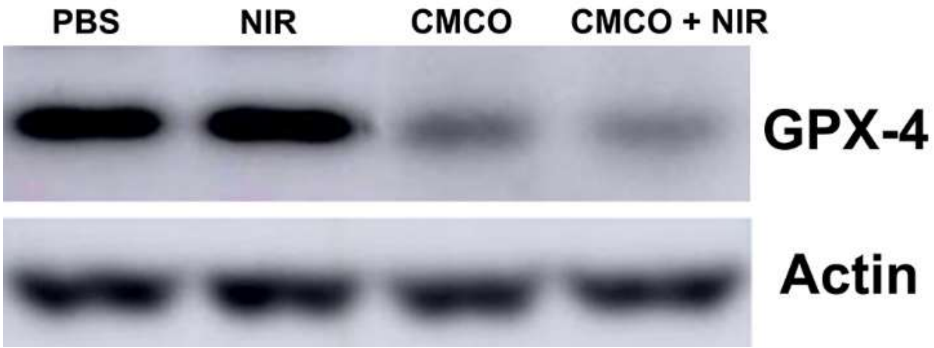

Figure S44. Western blot of FDX-1 after different treatments.

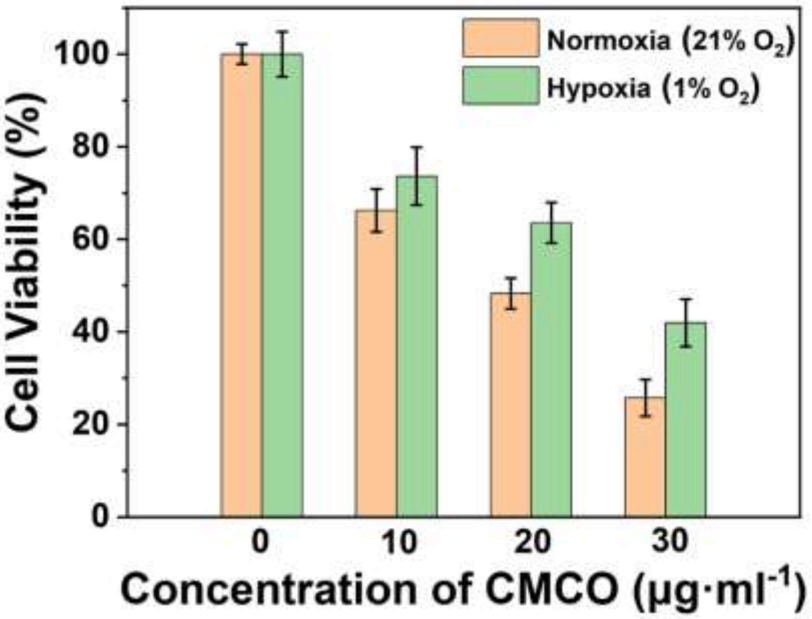

Figure S45. Cytotoxicity assessment on CT26 treated with different concentration of CMCO nanozymes upon normoxia (21% O_2) and hypoxia (1% O_2). Dates are presented as mean ± SD (n = 6).

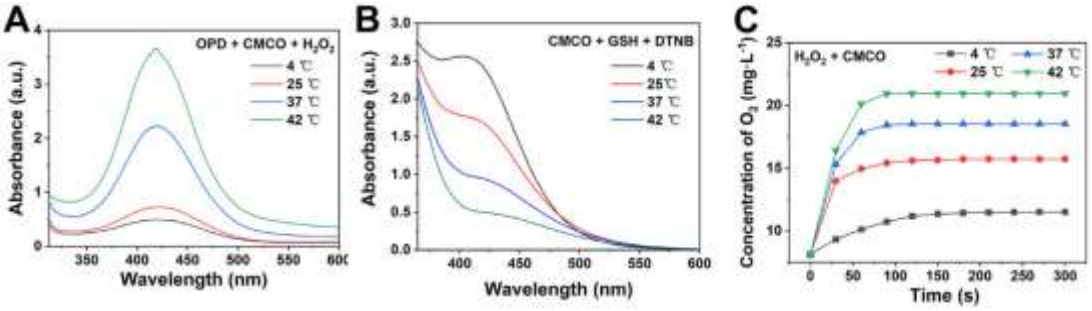

Figure S46. (A) ·OH generation curves of CMCO (20 μg·mL^{-1}) with OPD (1 mM) as a probe in the presence of H_2O_2 (1 mM) for 20 min at different temperature. (B) GSH consumption curves of CMCO (30 μg·mL^{-1}) with DTNB (100 μg·mL^{-1}) as a probe for 1 min at different temperature. (C) O_2 generation cures of CMCO (30 μg·mL^{-1}) in the presence of H_2O_2 (4 mM) at different temperature.

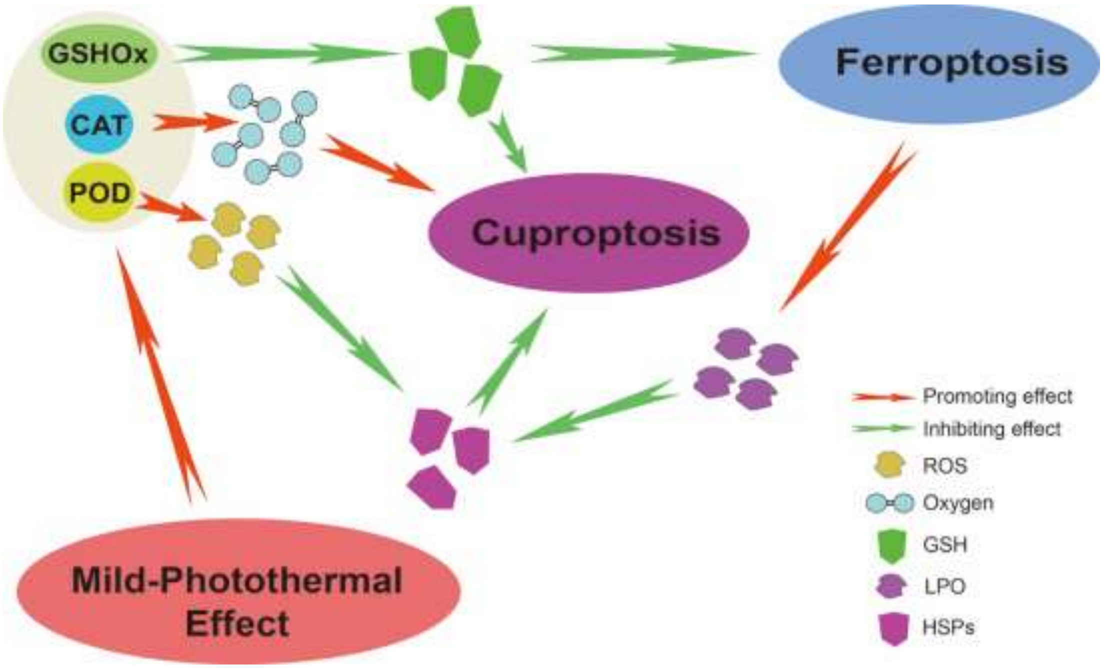

Figure S47. Schematic illustration of mild-photothermal effect induced high efficiency ferroptosis-boosted-cuproptosis based on CMC nanozyme.

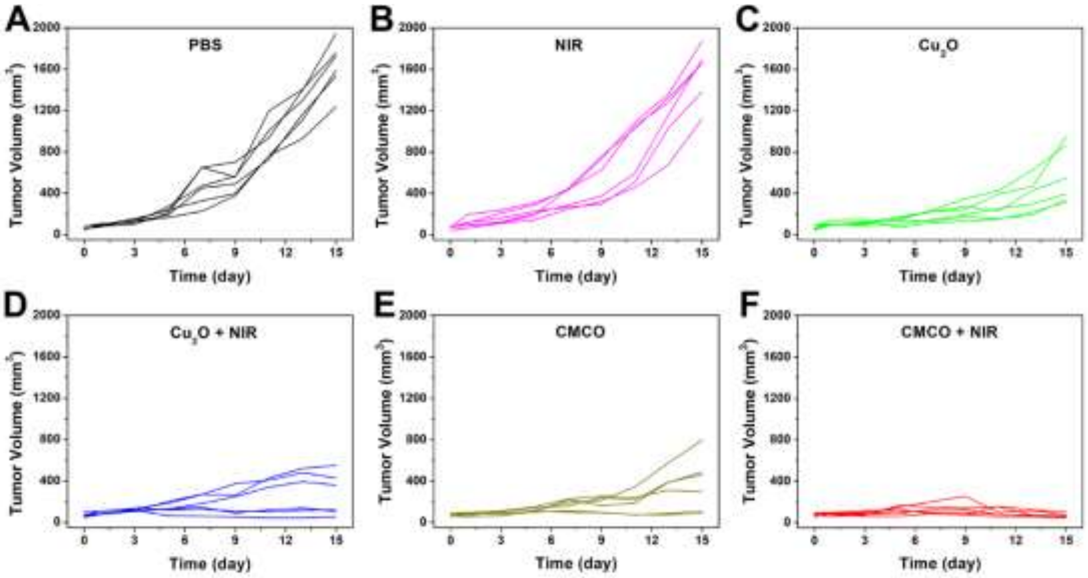

Figure S48. The tumor growth curves of CT26 tumor-bearing mice after different treatments with intratumor injection.

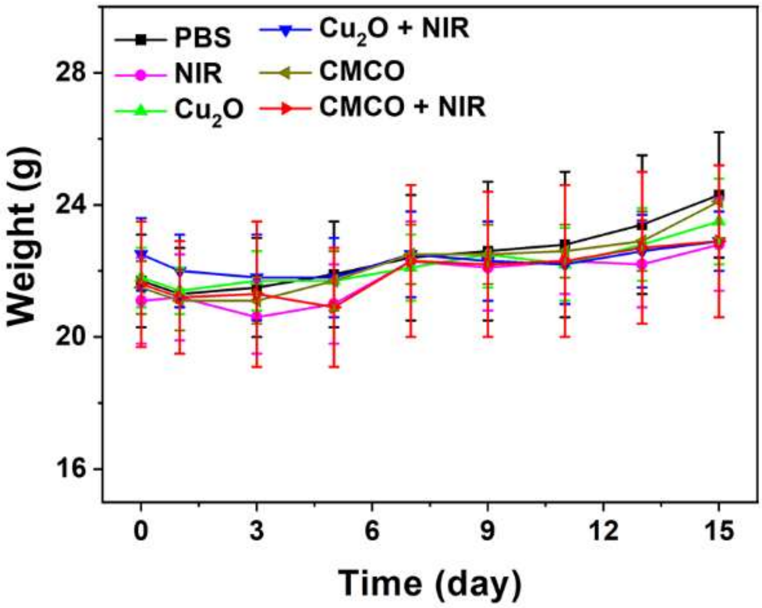

Figure S49. The body weights of CT26 tumor-bearing mice over time after different treatments with intratumor injection. Dates are presented as mean ± SD (n = 6).

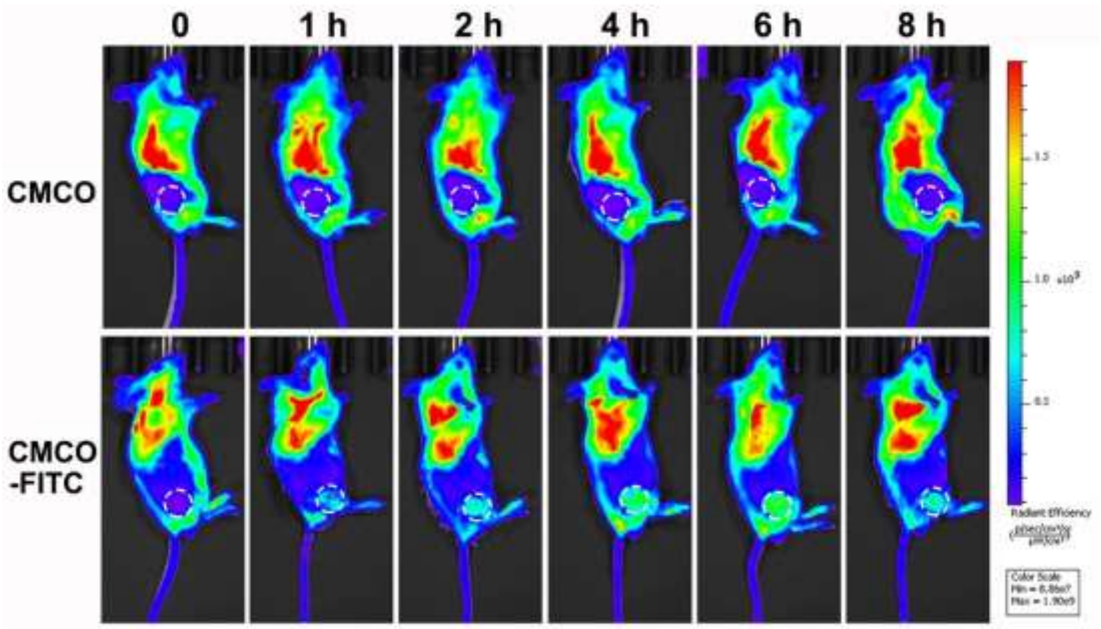

Figure S50. Fluorescence images of CMCO and CMCO-FITC accumulated in the tumor site of CT26-bearing mice injected via the tail vein.

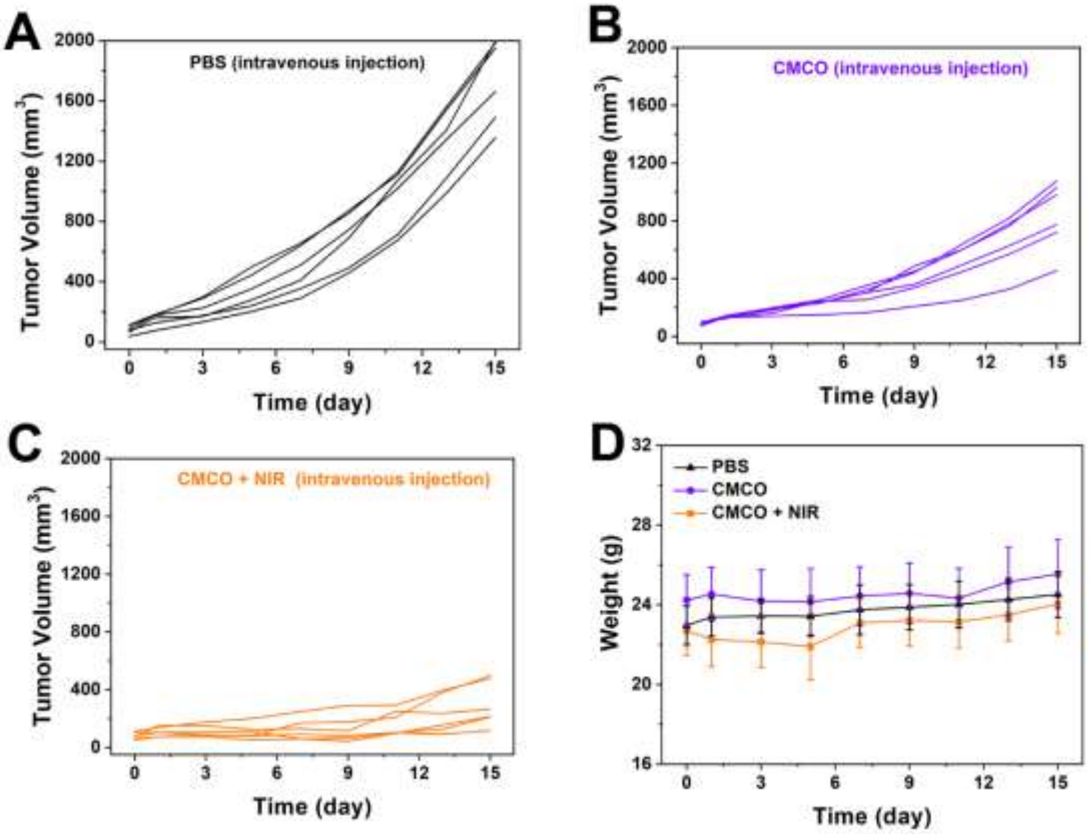

Figure S51. (A), (B)and (C) Tumor growth curves and (D) body weights of CT26 tumor-bearing mice after different treatments with intravenous injection. Dates in (D) are presented as mean ± SD (n = 6).

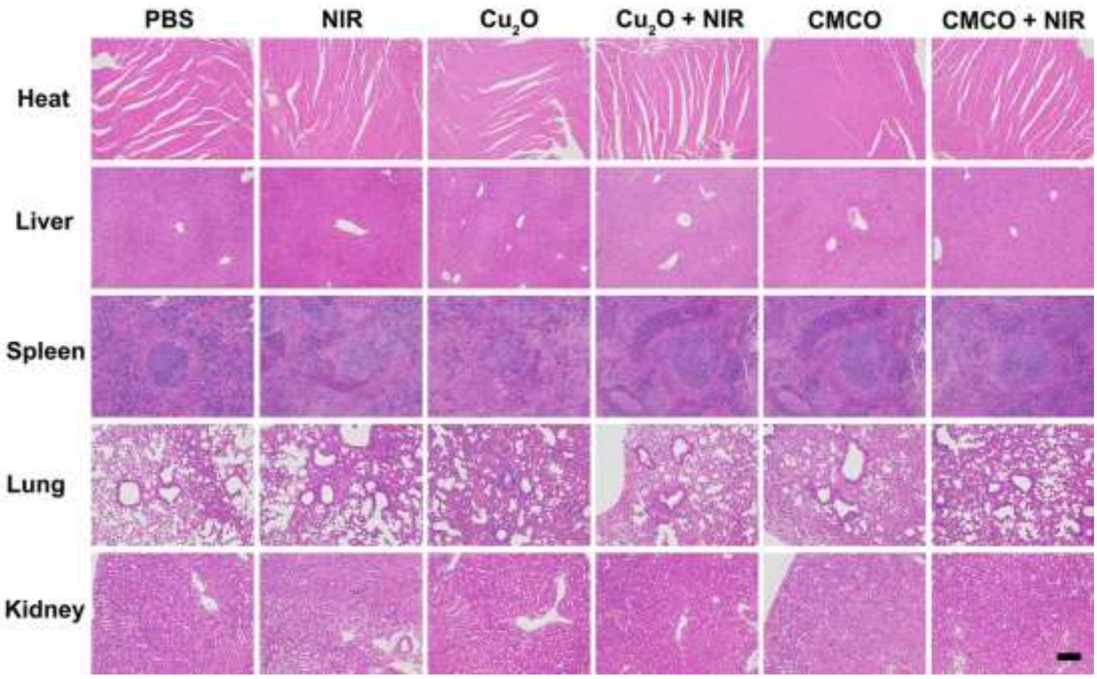

Figure S52. H&E staining of major organs after treated with PBS, NIR, Cu_2O, Cu_2O + NIR, CMCO, CMCO + NIR. Scar bar: 100 μm.

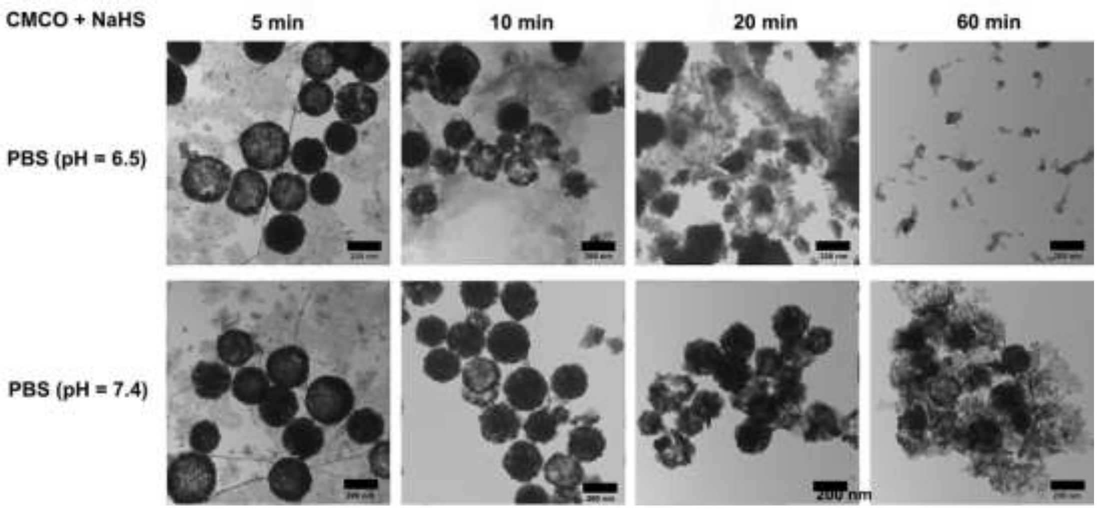

Figure S53. TEM images of CMCO nanozymes (100 μg·ml^{-1}) reacting with NaHS (200 μg·ml^{-1}) in PBS (pH = 6.5) and PBS (pH = 7.4) for 5, 10, 20, 60 min.

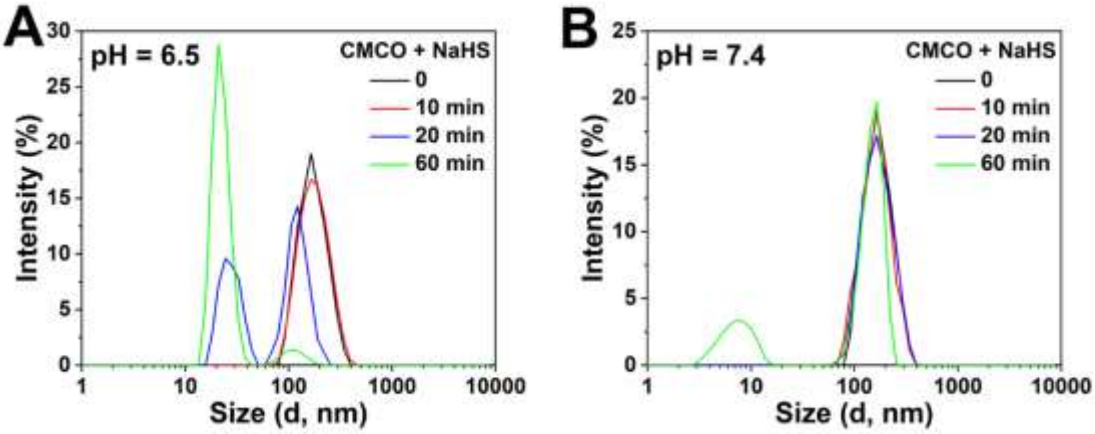

Figure S54. The size distribution of CMCO reacting with NaHS in PBS (pH = 6.5) and PBS (pH = 7.4) for different time (0, 10, 20 and 60 min).

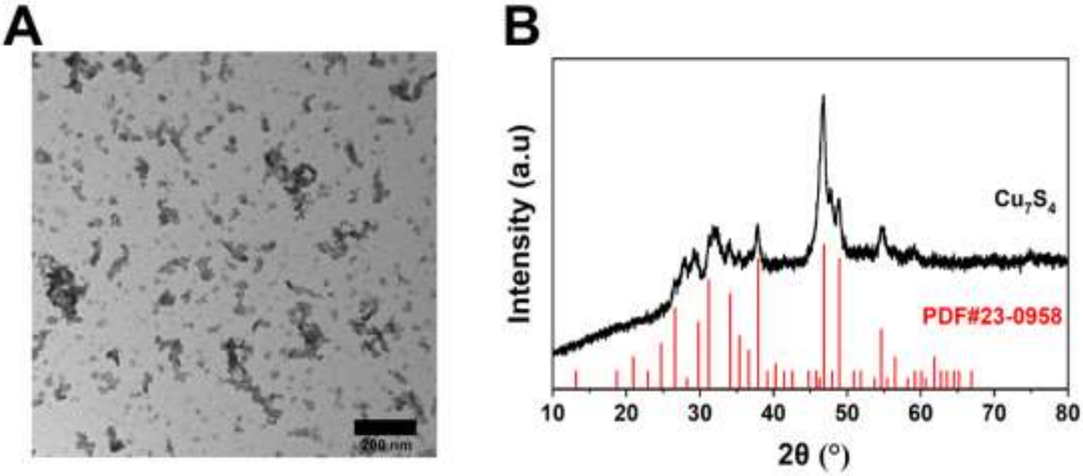

Figure S55. (A) TEM and (B) XRD of the products of Cu_2O reacting with NaHS for 1 min.

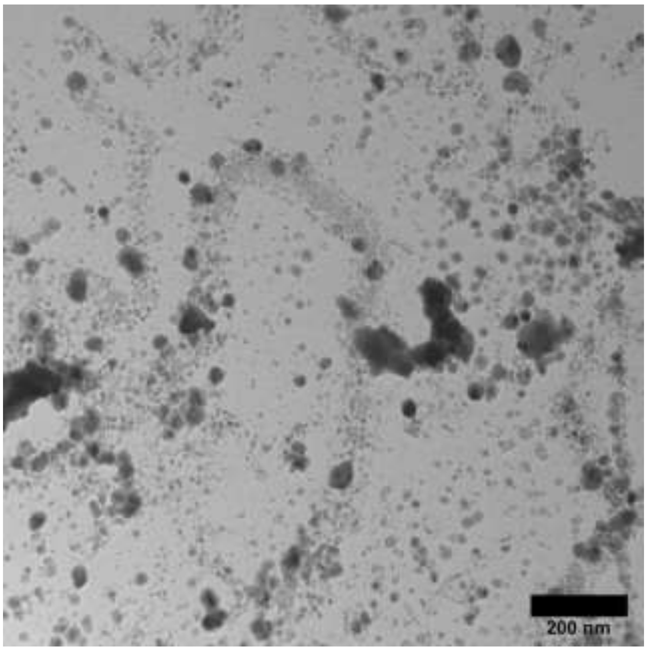

Figure S56. TEM of CMCO reacting with NaHS for 1 h in PBS (pH = 6.5).

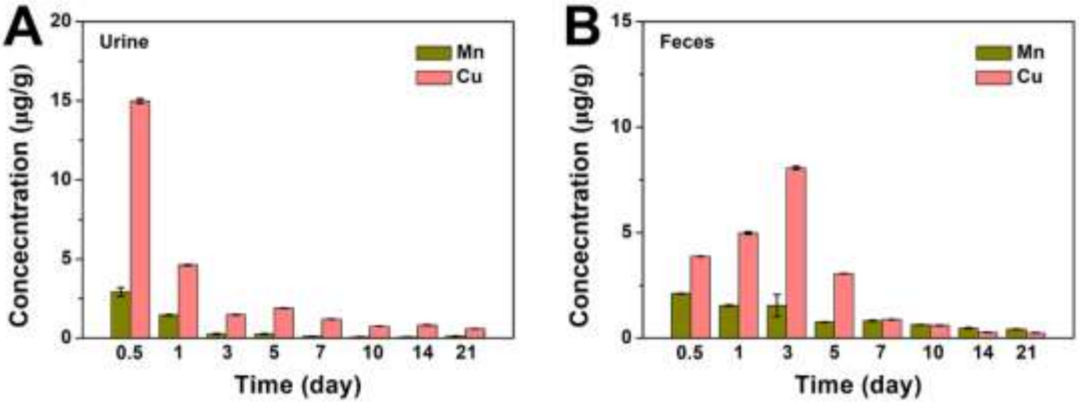

Figure S57. The metabolism of Mn and Cu from CT26 tumor-bearing mice through (A) urine and (B) feces after treated with CMCO + NIR. Dates are presented as mean ± SD (n = 3).

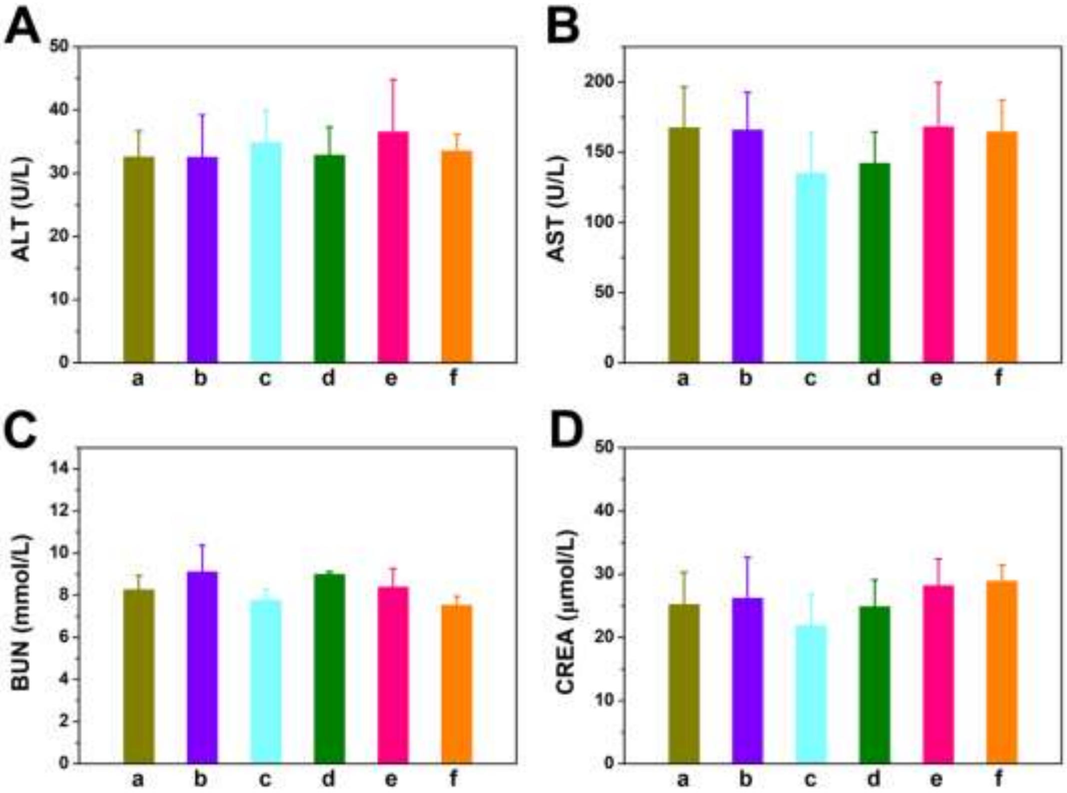

Figure S58. The biochemical parameters of liver and kidney after treated with (a) PBS, (b) NIR, (c) Cu_2O, (d) Cu_2O + NIR, (e) CMCO and (f) CMCO + NIR via intratumor injection. Dates are presented as mean ± SD (n = 3).
